# Efficacy, long-term survival and safety of different PD-1 inhibitors plus chemotherapy in recurrent or metastatic nasopharyngeal carcinoma: a systematic review and meta-analysis

**DOI:** 10.3389/fonc.2026.1849030

**Published:** 2026-06-15

**Authors:** Wen Sun, Yansha Liu, Hui Dong, Yun Huang

**Affiliations:** 1 Department of Otolaryngology, Yantaishan Hospital, Yantai, Shandong, China; 2 Department of Otolaryngology, Ganzhou People’s Hospital, Ganzhou, Jiang Xi, China

**Keywords:** chemotherapy, efficacy, meta−analysis, nasopharyngeal carcinoma, PD−1 inhibitor, survival

## Abstract

**Background:**

Recurrent or metastatic nasopharyngeal carcinoma (RM−NPC) is associated with poor prognosis. PD−1 inhibitors combined with chemotherapy have become the standard first−line treatment, but the comparative efficacy, long−term survival, and safety of different PD−1 inhibitors remain unclear. This study aimed to evaluate the impact of distinct PD−1 inhibitors plus chemotherapy for RM−NPC.

**Methods:**

We conducted a systematic review and single−arm meta−analysis including 19 studies with 1459 patients. Outcomes included objective response rate (ORR), disease control rate (DCR), progression−free survival (PFS), overall survival (OS), and grade ≥3 treatment−related adverse events (TRAEs). Pooled analyses and subgroup comparisons stratified by PD−1 inhibitor type were performed.

**Results:**

The pooled ORR was 64% and DCR was 94% across all regimens. Subgroup analyses stratified by PD−1 inhibitor type revealed significant differences: toripalimab−based regimens achieved the highest ORR and longest median PFS of 21.4 months, while tislelizumab plus chemotherapy yielded the most favorable median OS of 45.3 months. Camrelizumab plus chemotherapy showed a median OS of 34.5 months. Subgroup analysis by study design (randomized controlled trials vs. non−randomized single−arm studies) confirmed highly consistent outcomes, supporting the robustness of pooled estimates. The pooled incidence of grade ≥3 TRAEs was 13%, with comparable safety profiles across all PD−1 inhibitors and no unexpected safety signals.

**Conclusions:**

PD−1 inhibitors combined with chemotherapy provide robust efficacy and durable long−term survival benefits in RM−NPC. Toripalimab, tislelizumab, and camrelizumab demonstrate superior therapeutic effects compared with other agents, with toripalimab showing optimal tumor response and PFS, and tislelizumab offering the best long−term OS. The findings support individualized PD−1 inhibitor selection in clinical practice and confirm the reliability of single−arm meta−analyses combining randomized and non−randomized evidence for agent−stratified comparison.

**Systematic Review Registration:**

https://www.crd.york.ac.uk/prospero/, identifier CRD420261361323.

## Introduction

Nasopharyngeal carcinoma (NPC) is an Epstein–Barr virus (EBV)-associated epithelial malignancy with a striking geographic disparity, disproportionately affecting populations in southern China, Southeast Asia, and North Africa (1, 2). Despite advances in radiotherapy and systemic therapy for locoregional disease, approximately 20–30% of patients eventually develop recurrent or metastatic NPC (RM-NPC), a condition associated with a dismal prognosis and limited long-term survival (3, 4). For decades, platinum-based chemotherapy has served as the first-line standard of care for RM-NPC, yet median overall survival (OS) rarely exceeds 2 years, and treatment-related toxicities remain a significant clinical burden (5).

The advent of programmed cell death-1 (PD-1) inhibitors has transformed the therapeutic landscape of RM-NPC. Multiple landmark phase 3 randomized controlled trials (RCTs), including CAPTAIN-1st, JUPITER-02, and RATIONALE-309, have consistently demonstrated that PD-1 inhibitors combined with gemcitabine plus platinum chemotherapy significantly improve progression-free survival (PFS) and OS compared with chemotherapy alone, establishing chemo-immunotherapy as the new first-line standard (6–8). However, a growing number of PD-1 inhibitors (approved globally or in China) have been approved for RM-NPC, creating a critical clinical gap: direct head-to-head comparisons between different agents are lacking, and existing systematic reviews rarely comprehensively synthesize efficacy, long-term survival, and safety stratified by individual PD-1 inhibitors.

Several recent meta-analyses have evaluated PD-1 inhibitors plus chemotherapy in RM-NPC (9–11). However, these studies either lacked agent-stratified long-term survival data, did not separate RCTs from observational studies, or included fewer phase III trials with mature follow-up. The present study provides the most comprehensive agent-stratified synthesis of efficacy, long-term survival, and safety, with separate analysis of RCTs and non-randomized studies, and updated mature OS data from latest phase III trials. Given the rising clinical demand for personalized treatment selection, a comprehensive synthesis of comparative performance across PD-1 inhibitors is urgently needed to guide clinical decision-making. Therefore, we conducted this systematic review and single-arm meta-analysis to evaluate the efficacy, long-term survival, and safety of different PD-1 inhibitors plus chemotherapy in patients with RM-NPC, with subgroup analyses stratified by PD-1 inhibitor type. Our findings aim to provide high-level evidence to optimize first-line therapeutic strategies for RM-NPC.

## Materials and methods

### Study eligibility criteria

This systematic review and meta-analysis was conducted and reported in strict adherence to the Preferred Reporting Items for Systematic Reviews and Meta−Analyses (PRISMA 2020) guidelines (12). The study protocol was prospectively registered in the international prospective register of systematic reviews (PROSPERO) with the registration number CRD420261361323. Eligible studies were selected based on the following predefined inclusion and exclusion criteria. Deviations from the registered protocol are explicitly disclosed: (1) The protocol initially planned to include only non-randomized studies, but both RCTs and non-randomized single-arm studies were finally included to ensure comprehensiveness of evidence; (2) The protocol mentioned Cochrane ROB-1, ROB-2, NOS for quality assessment, while Methodological Index for Non−Randomized Studies (MINORS) was used for non-randomized studies and RoB 2 for RCTs in this study; (3) The protocol initially restricted to English publications, but no language restriction was applied in the final search to avoid missing eligible evidence.

### Search strategy and data sources

A systematic literature search was performed across four major electronic databases, including PubMed, Embase, Cochrane Library, and Web of Science, from the date of database establishment to March 2026. The search strategy combined free-text terms related to nasopharyngeal carcinoma, recurrent/metastatic disease, PD−1 inhibitors, immunotherapy, and chemotherapy. No language or publication status restrictions were applied during the initial search phase. Additionally, we manually screened the reference lists of all included studies and relevant systematic reviews to identify any potentially eligible publications that had not been captured by the database searches. Full search strategies for PubMed, Embase, Cochrane Library, and Web of Science are provided in **Supplementary Table 1**. The main reasons for exclusion after screening were: wrong study population, wrong intervention, no available outcome data, review/editorial letters, and duplicate publications.

### Study selection process

Studies selection was conducted independently by two trained reviewers following a three-step procedure. First, duplicate records were removed using reference management software. Second, all retrieved records were initially screened based on titles and abstracts to exclude irrelevant studies. Third, full-text articles of the remaining studies were retrieved and evaluated for final inclusion. Any discrepancies or disagreements regarding study eligibility were resolved through consensus discussion; if consensus could not be reached, a third senior reviewer was consulted to make the final decision. A PRISMA flow diagram was constructed to systematically document the entire study selection process.

### Data extraction

Standardized data extraction forms were designed and used by two independent reviewers to extract key data from the included studies. The extracted data included basic study characteristics (first author, publication year, study design, sample size, patient age, and gender distribution), intervention details (type of PD−1 inhibitor and chemotherapeutic regimen), and outcome data. Outcome data covered objective tumor response measures (complete response, CR; partial response, PR; objective response rate, ORR; disease control rate, DCR), long−term survival outcomes (progression−free survival, PFS; overall survival, OS; and 1−, 2−, 3−, and 5−year survival rates), and safety profiles (incidence of grade ≥3 treatment−related adverse events, TRAEs). For continuous survival outcomes, median values and corresponding 95% confidence intervals (CIs) or ranges were extracted. For binary outcomes, the number of events and total number of patients were recorded. Disagreements during data extraction were resolved through discussion, with a third reviewer providing adjudication if necessary.

### Quality assessment of included studies

The methodological quality of the included non−randomized single−arm studies was evaluated using the MINORS scale. The MINORS scale comprises 8 domains, each scored from 0 to 2, with a maximum total score of 16. Each item was rated as 0 (not reported), 1 (reported but inadequate), or 2 (reported and adequate). Based on their total MINORS scores, included studies were classified into three quality levels: high quality (score ≥13), moderate quality (score 9–12), and low quality (score <9). Low−quality studies were excluded from the final quantitative synthesis. RCTs were evaluated using the Cochrane RoB 2 tool, in line with methodological standards. Two reviewers independently performed the quality assessment, and any inconsistencies were resolved by consensus or by a third reviewer.

### Statistical analysis

Statistical analyses were performed using Review Manager 5.4. Given the single-arm study design of the included trials, a single-arm rate meta-analysis was implemented to synthesize the outcome data. For binary outcomes, including ORR, DCR, CR, PR, Stable disease (SD), 1−/3−/5−year survival rates, and incidence of grade ≥3 TRAEs, the pooled proportions with 95% CIs were calculated. For continuous survival outcomes, including median PFS and median OS, the pooled median values with 95% CIs were synthesized. Heterogeneity across studies was assessed using the Cochran Q test and the I² statistic. An I² value <50% was considered to indicate low heterogeneity, and a fixed-effects model (Mantel–Haenszel method) was used for data synthesis. An I² value ≥50% indicated significant heterogeneity, and a random-effects model was applied. A two-sided P value <0.05 was considered statistically significant. Subgroup analysis stratified by study design (RCTs vs. non-randomized single-arm studies) was performed to assess the robustness of pooled estimates and explore potential design-related heterogeneity.

## Results

### Literature search and study characteristics

**Figure 1:**
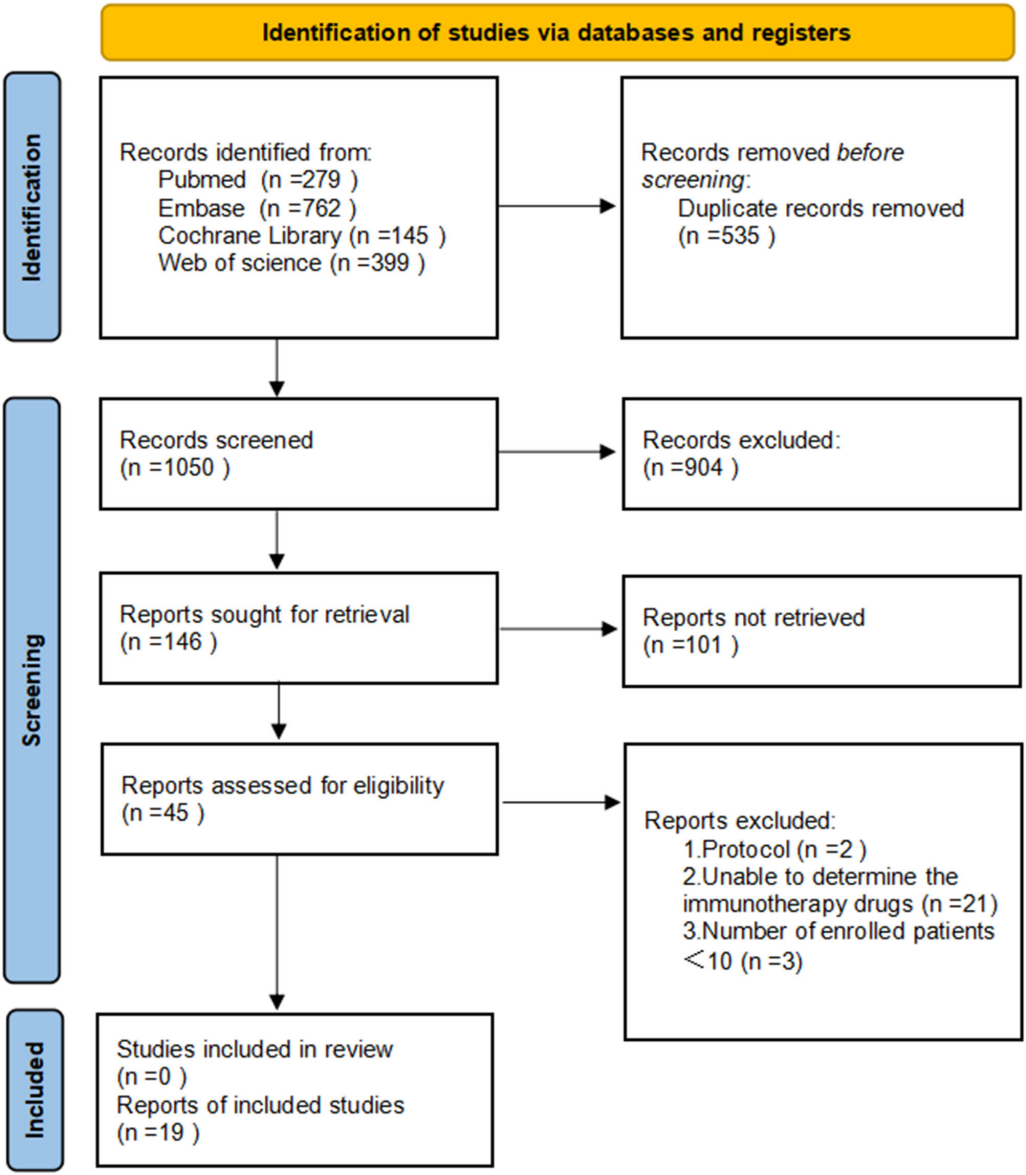
PRISMA flow diagram of study selection.

A comprehensive literature search was conducted in PubMed, Embase, Cochrane Library, and Web of Science up to March 2026. After removing duplicates, a total of 1050 records were screened by title and abstract. Then, 45 full-text articles were assessed for eligibility. Finally, 19 studies (6, 13–30) met the predefined inclusion criteria and were included in this systematic review and meta−analysis (**Figure 1**).

The basic characteristics of all included studies are summarized in **Tables 1**, **2**. Collectively, these studies enrolled a total of 1459 patients with R/M−NPC. The included studies covered a variety of PD−1 inhibitors, including camrelizumab, tislelizumab, toripalimab, nivolumab, serplulimab, penpulimab, as well as immune−targeted bispecific antibodies such as QL1706 (a combination of iparomlimab and tuvonralimab targeting PD-1 and CTLA-4), cadonilimab, tagitanlimab, and the combination regimen of LBL−007 (targeting LAG-3) plus tislelizumab. Chemotherapy backbone regimens mainly consisted of gemcitabine plus cisplatin/carboplatin, cisplatin plus fluorouracil, gemcitabine alone, and other platinum−based doublet or triplet regimens. Study designs included phase I, phase II, phase III, single−arm, multicenter, randomized, double−blind, and prospective studies, covering both controlled trials and single−arm observational studies.

**Table 1:** Main characteristics of included studies.

Author year	NCT number/ethical registration number	Study design	Immune drugs	Chemotherapy drugs	No. of patient	Median age	Sex(M/Fe)
Wenfeng Fang2018 (13)	NCT 03121716	Single-arm, Phase I	Camrelizumab	Gemcitabine + Cisplatin	23	44 (34-51 )	17/6
Yunpeng Yang2021 (6)	NCT03707509	Multicenter, Randomized, Double-blind, Phase III	Camrelizumab	Gemcitabine + Cisplatin	134	49-52	113/21
X. Wang2022 (14)	–	Prospective study	Tislelizumab	low-dose Gemcitabine + Capecitabine	25	–	–
Rui You2022 (15)	NCT04073784	Single-arm, Prospective, Phase II	Toripalimab	Gemcitabine + Apatinib	41	46	31/10
Hyun Ae Jung2022 (16)	–	Single-arm, Prospective, Phase II	Nivolumab	Gemcitabine	36	–	–
Si-Yuan Chen2023 (17)	NCT04398056	Single-arm, Phase II	Toripalimab	Cisplatin + Fluorouracil (PF)	22	54.5	15/7
Hai-Qiang Mai2023 (18)	NCT03581786	International multicenter, Randomized, Double-blind, Phase III	Toripalimab	Gemcitabine + Cisplatin	146	46 (38-53)	1224/22
L. Zhang2024 (19)	NCT05513573	Multicenter, Randomized, Double-blind, Phase II	Serplulimab	Gemcitabine + Cisplatin ± HLX07 (anti-EGFR)	75	–	–
Shuang Huang2024 (20)	NCT04736810	Prospective, Single-arm, Phase II	Penpulimab	Anlotinib + Gemcitabine (GAP regimen)	14	–	–
Yan Huang2024 (21)	NCT05576272	Multicenter, Single-arm, Phase II (DUBHE-N302)	QL1706 (PD-1/CTLA-4 bispecific antibody)	Gemcitabine + Cisplatin	29	50 (25-65 )	24/5
Xiong Zou2024 (22)	NCT04405622	Single-center, Single-arm, Phase II	Toripalimab	Gemcitabine	21	55 (34-70)	16/5
Yaofei Jiang2025 (23)	ChiCTR2200067057	Single-center, Single-arm, Phase II	Cadonilimab	TPC Chemotherapy (Albumin-bound Paclitaxel + Cisplatin / Lobaplatin + Capecitabine)	25	44 (24-60)	21/4
Jingjing Miao2025 (24)	NCT03930498	Prospective, Single-arm, Phase II	Toripalimab	Gemcitabine + Cisplatin	68	52 (43.0-58.0)	51/17
Yuankai Shi2025 (25)	NCT05294172	Multicenter, Randomized, Double-blind, Phase III	Tagitanlimab (KL-A167)	Gemcitabine + Cisplatin	197	52	157/40
Cheng Xu2025 (26)	NCT03984357	Multicenter, Single-arm, Phase II	Nivolumab	Gemcitabine	152	49 (18-65)	124/28
Chaosu Hu2025 (27)	NCT04974398	Multicenter, Randomized, Double-blind, Phase III	Penpulimab	chemotherapy	144	–	–
Yan Huang2026 (28)	NCT03707509	Randomized, Double-blind, Phase III (CAPTAIN-1st)	Camrelizumab	Gemcitabine + Cisplatin	134	49	–
Dongchen Sun2026 (29)	NCT05516914	Multicenter, Single-arm, Phase II	LBL-007 (anti-LAG-3) + Tislelizumab	Gemcitabine + Cisplatin	42	50 (29-71)	33/9
Yunpeng Yang2026 (30)	NCT03924986	Multicenter, Randomized, Double-blind, Phase III (RATIONALE-309)	Tislelizumab	Gemcitabine + Cisplatin	131	50.0 (26-74 )	103/28

**Table 2:** Main characteristics of included studies.

Author year	CR	PR	ORR	SD	DCR	Median PFS (month)	Median OS (month)	1-year PFS rate	2-year PFS rate	3-years PFS rate	1-year OS rate	3-year OS rate	5-year OS rate	≥3TRAEs
Wenfeng Fang2018 (13)	1/22	19/22	20/22	2/22	22/22	10.2 (9.7-10.8)	–	13/22	–	–	–	–	–	20/23
Yunpeng Yang2021 (6)	7/134	110/134	117/134	12/134	129/134	10.8 (8.5-13.6)	–	–	–	–	–	–	–	124/134
X. Wang2022 (14)	5/25	12/25	17/25	5/25	22/25	–	–	18/23	–	–	–	–	–	–
Rui You2022 (15)	14/41	23/41	37/41	4/41	41/41	25.8	–	–	21/41	–	39/41		–	23/41
Hyun Ae Jung2022 (16)	–	–	13/36	23/36	35/36	13.8 (8.6-16.8 )	–	–	–	–			–	–
Si-Yuan Chen2023 (17)	5/22	13/22	18/22	–	18/22	–	–	–	–	10/22	14/22	10/22	–	15/22
Hai-Qiang Mai2023 (18)	39/146	76/146	115/146	18/146	133/146	21.4 (14.1-NR)	–	–	–	–	133/146	94/146	–	131/146
L. Zhang2024 (19)	–	–	54/75	–	–	–	–	40/75	–	–	–	–	–	14/75
Shuang Huang2024 (20)	1/13	11/13	12/13	–	–	–	–	–	–	–	–	–	–	11/13
Yan Huang2024 (21)	1/29	23/29	24/29	4/29	28/29	12.5 (5.7-NE)	–	–	–	–	–	–	–	18/29
Xiong Zou2024 (22)	3/21	10/21	13/21	8/21	21/21	11.8 (5.6-18.0)	–	–	–	–	20/21	–	–	5/21
Yaofei Jiang2025 (23)	3/25	14/25	17/25	6/25	23/25	10.6 (5.2-16.0)	–	–	–	–	19/25	–	–	12/25
Jingjing Miao2025 (24)	–	44/68	44/68	22/68	66/68	–	–	–	33/68	–	–	–	–	21/68
Yuankai Shi2025 (25)	–	–	161/197	–	–	–	–	–	–	–	–	–	–	114/197
Cheng Xu2025 (26)	23/152	111/152	134/152	12/152	146/152	–	–	–	–	134/152	–	149/152	–	61/152
Chaosu Hu2025 (27)	–	–	98/144	–	–	9.6 (7.1- 12.5)	–	–	–	–	–	–	–	128/144
Yan Huang2026 (28)	–	–	–	–	–	–	34.5 (29.4-45.7)	–	–	–	–	–	99/263	–
Dongchen Sun2026 (29)	1/42	34/42	35/42	6/42	41/42	15.8 (9.9-NE)	–	–	–	–	36/42	–	–	37/42
Yunpeng Yang2026 (30)	21/131	70/131	91/131	19/131	110/131	9.6 (7.6-11.6)	45.3 (33.4-NE)	–	–	–	117/131	73/131	–	106/131

CR, Complete response; PR, Partial response; SD, Stable disease; ORR, Objective response rate; DCR, Disease control rate; PFS:Progression-Free Survival; OS, Overall Survival; TRAEs, Treatment-related adverse event.

### Quality assessment of included studies

Results showed that all 12 non-randomized single-arm studies achieved scores ≥11, with 9 studies scoring 15–16 (high quality) and 3 studies scoring 11–14 (moderate to high quality); no study was rated as low quality. Common limitations included insufficient reporting of follow-up duration, unclear loss to follow-up, and lack of prospective sample size calculation (**Supplementary Table 2**).

Among 7 included RCTs, six were assessed as overall low risk of bias, and one phase II RCT was rated as some concerns due to incomplete reporting of follow-up data and subgroup analyses. No RCT was judged to have high risk of bias in any domain. The distribution and summary of risk of bias for RCTs are illustrated in **Supplementary Figure 1** and **Supplementary Figure 2**, respectively. Overall, the included studies exhibited acceptable to high methodological quality, supporting the reliability of the synthesized evidence.

### Subgroup analysis by study design

Subgroup analysis stratified by study design (RCTs vs. non-randomized single-arm studies) was performed to assess the robustness of pooled estimates and explore potential design-related heterogeneity. Pooled proportions for ORR, DCR, and grade ≥3 TRAEs, as well as pooled median values for PFS and OS, were calculated separately in each subgroup. Heterogeneity was evaluated using the I^2^ statistic and Cochran Q test.

Results showed that pooled efficacy and safety outcomes were highly consistent between the RCT subgroup and non-randomized single-arm subgroup, with no statistically significant between-subgroup differences observed for any key endpoint. Within-group heterogeneity was low to moderate in both subgroups, and between-subgroup heterogeneity was minimal. These findings confirmed that study design did not significantly influence the overall pooled results, supporting the reliability and robustness of the main analysis (**Supplementary Figures 3**–**11**).

### Efficacy outcomes

**Figure 2:**
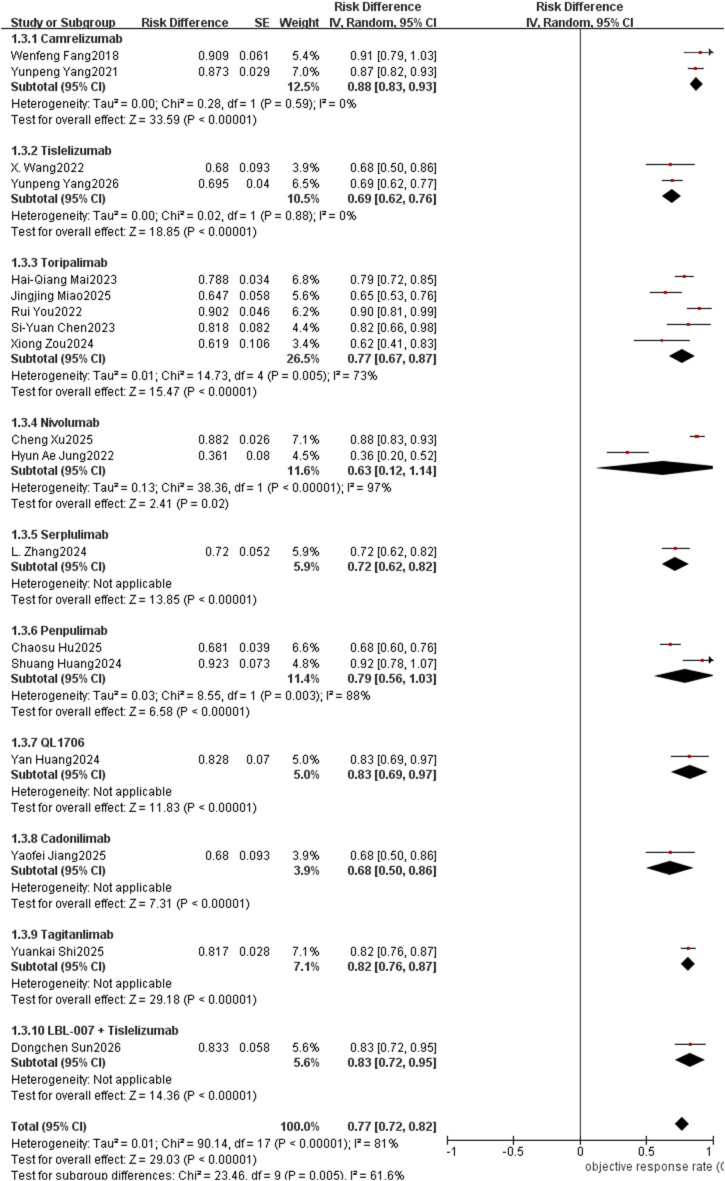
Forest plot of objective response rate (ORR) stratified by different PD−1 inhibitors in the single-arm meta-analysis, presented as pooled proportion.

Tumor response was evaluated using CR, PR, ORR, SD, and DCR. Data for ORR were available from all 18 studies enrolling 1325 patients. The pooled ORR of PD−1 inhibitors plus chemotherapy in R/M−NPC was robust, with noticeable differences across distinct immune checkpoint inhibitors (**Figure 2**). Among them, toripalimab−based regimens achieved relatively high ORR, while camrelizumab, tislelizumab, and nivolumab also exhibited favorable anti−tumor activity.

**Figure 3:**
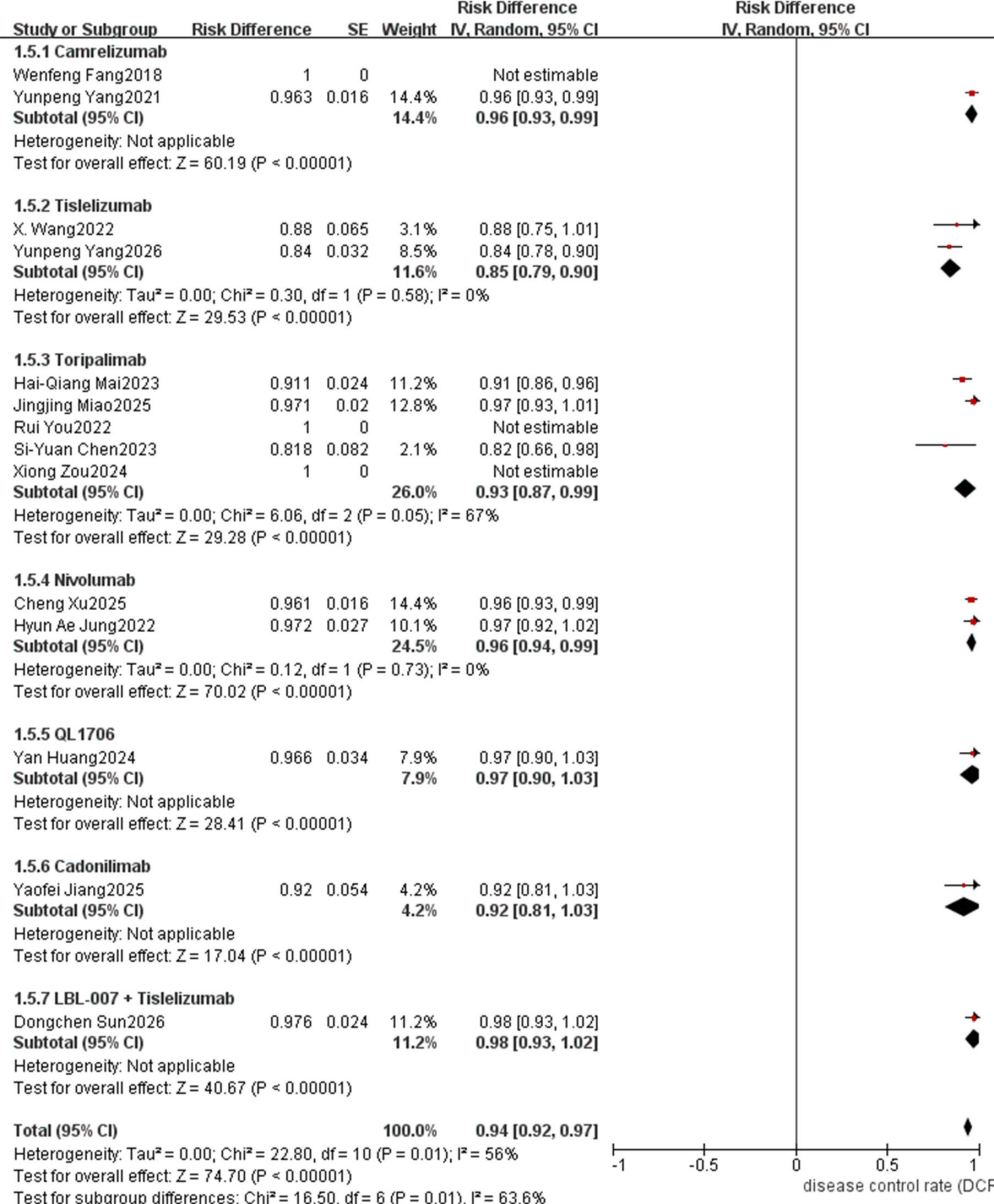
Forest plot of disease control rate (DCR) in patients treated with PD−1 inhibitors plus chemotherapy in the single-arm meta-analysis, presented as pooled proportion.

**Figure 4:**
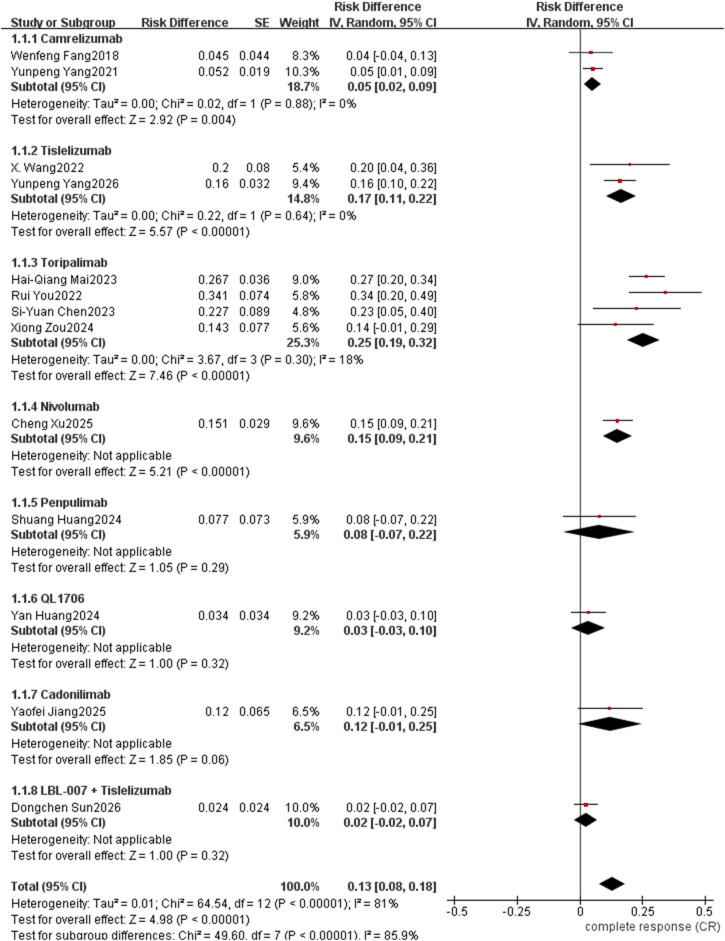
Forest plot of complete response (CR) rate stratified by PD−1 inhibitor type in the single-arm meta-analysis, presented as pooled proportion.

**Figure 5:**
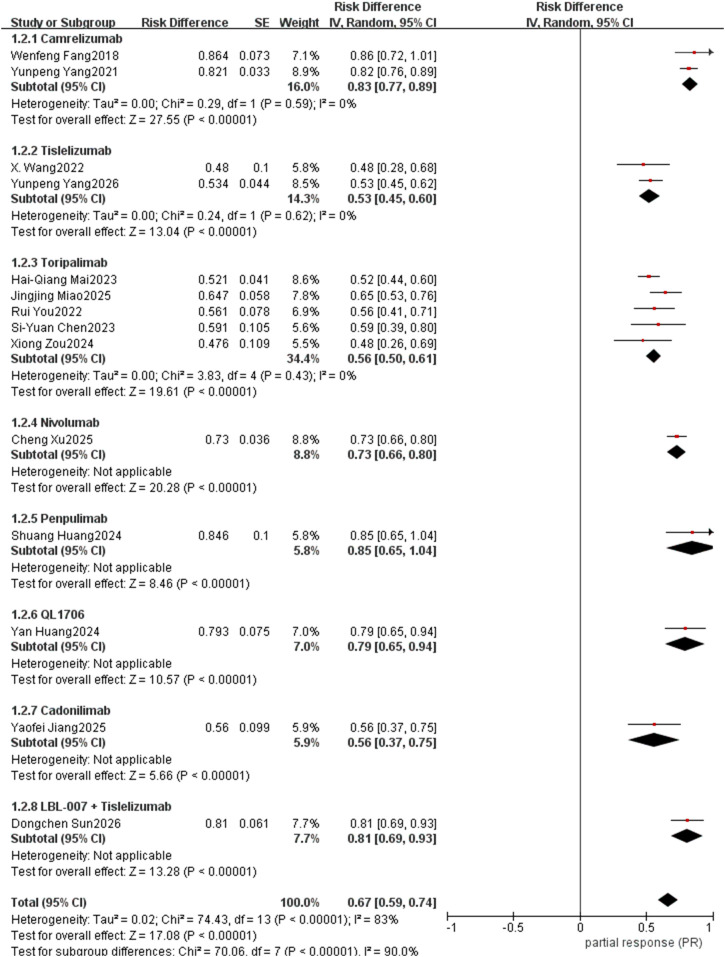
Forest plot of partial response (PR) rate in all included studies in the single-arm meta-analysis, presented as pooled proportion.

**Figure 6:**
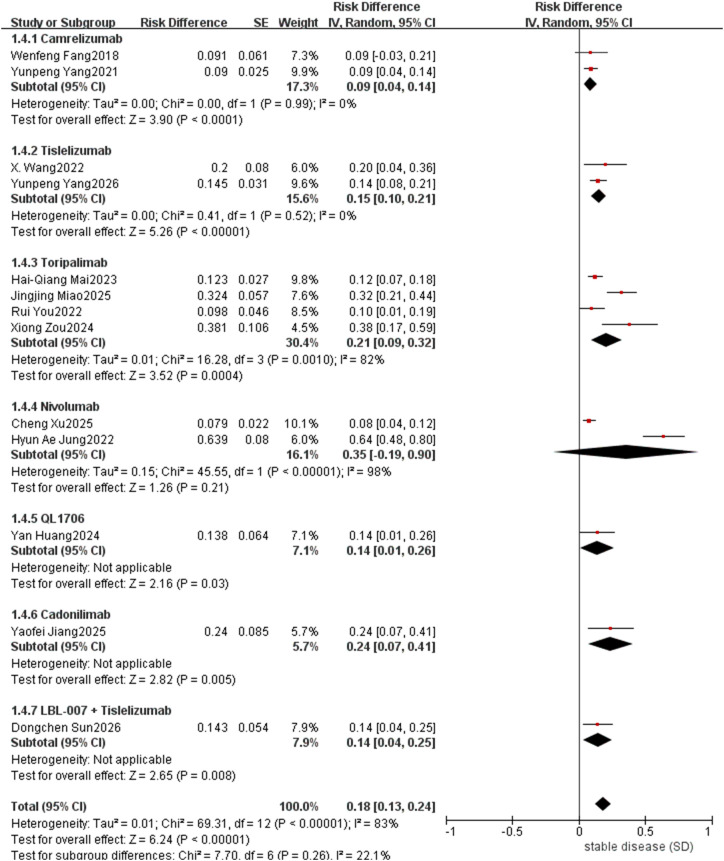
Forest plot of stable disease (SD) rate in all included studies in the single-arm meta-analysis, presented as pooled proportion.

For DCR, 15 studies provided available data, and the pooled DCR remained at a high level, indicating satisfactory short−term tumor control (**Figure 3**). The rates of CR and PR varied across different agents and study designs. Phase III trials of toripalimab and camrelizumab showed considerable CR proportions, whereas phase II single−arm studies presented relatively dispersed PR values (**Figures 4**, **5**). SD was reported in a substantial proportion of patients, contributing to the overall high DCR (**Figure 6**). Significant heterogeneity was detected across studies, which could be partly attributed to the type of PD−1 inhibitor, chemotherapy backbone, sample size, and study phase.

### Long−term survival outcomes

Long−term survival outcomes including median PFS, median OS, and survival rates at different time points were synthesized. Median PFS data were available from 11 studies, and the pooled median PFS ranged from 9.6 to 25.8 months across different regimens. Notably, the toripalimab plus gemcitabine and cisplatin regimen achieved the longest median PFS of 21.4 months, while camrelizumab and tislelizumab combinations provided stable and favorable PFS benefits.

**Figure 7:**
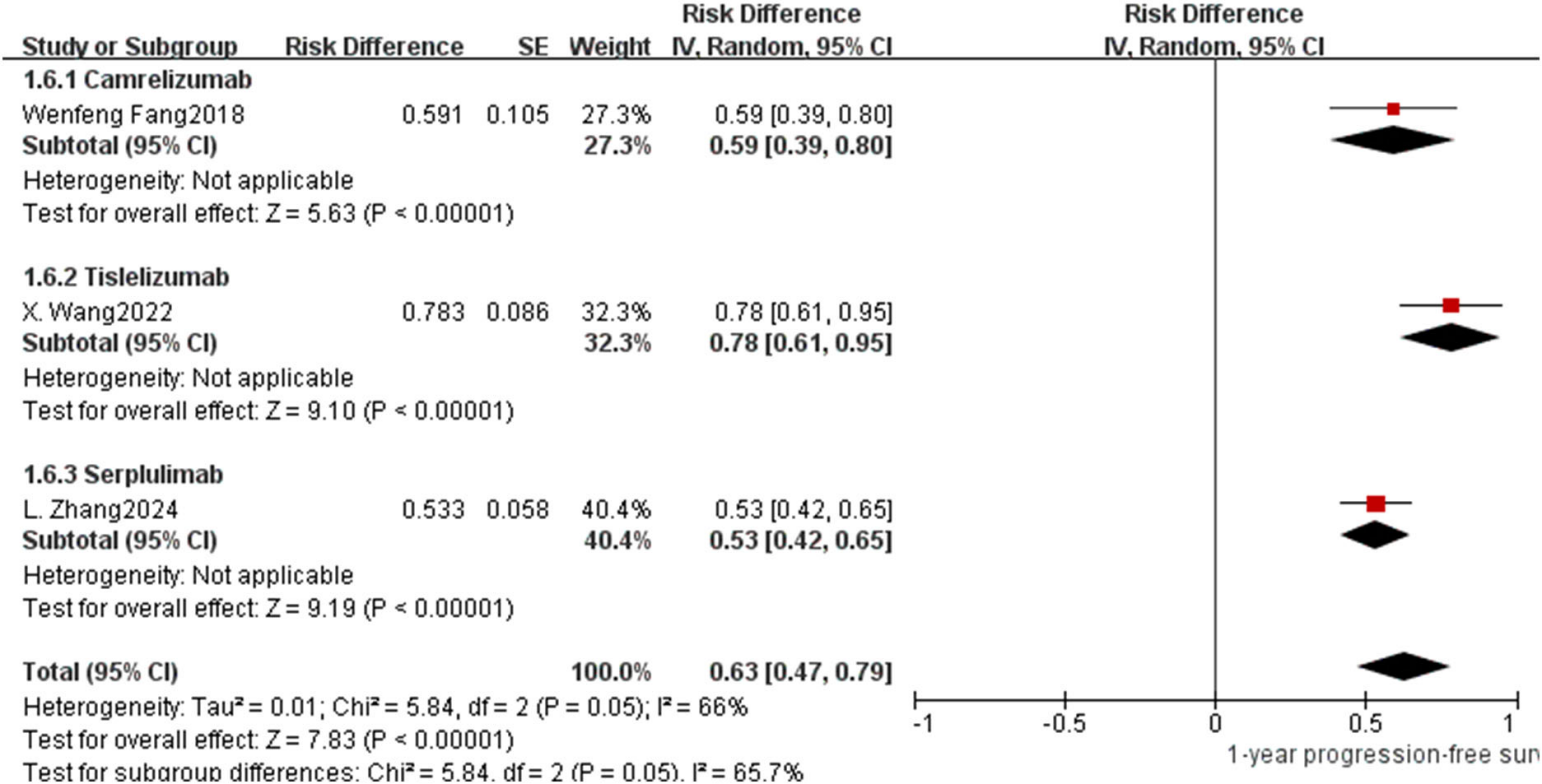
Forest plot of 1−year progression−free survival (PFS) rate in the single-arm meta-analysis, presented as pooled proportion.

**Figure 8:**
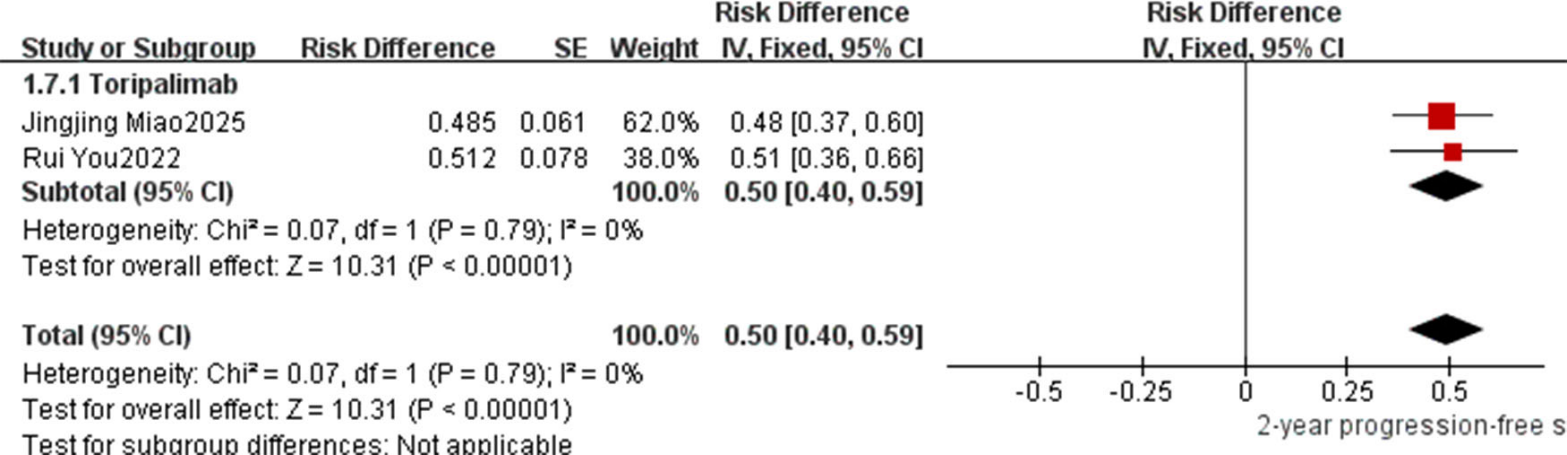
Forest plot of 2−year progression−free survival (PFS) rate in the single-arm meta-analysis, presented as pooled proportion.

**Figure 9:**
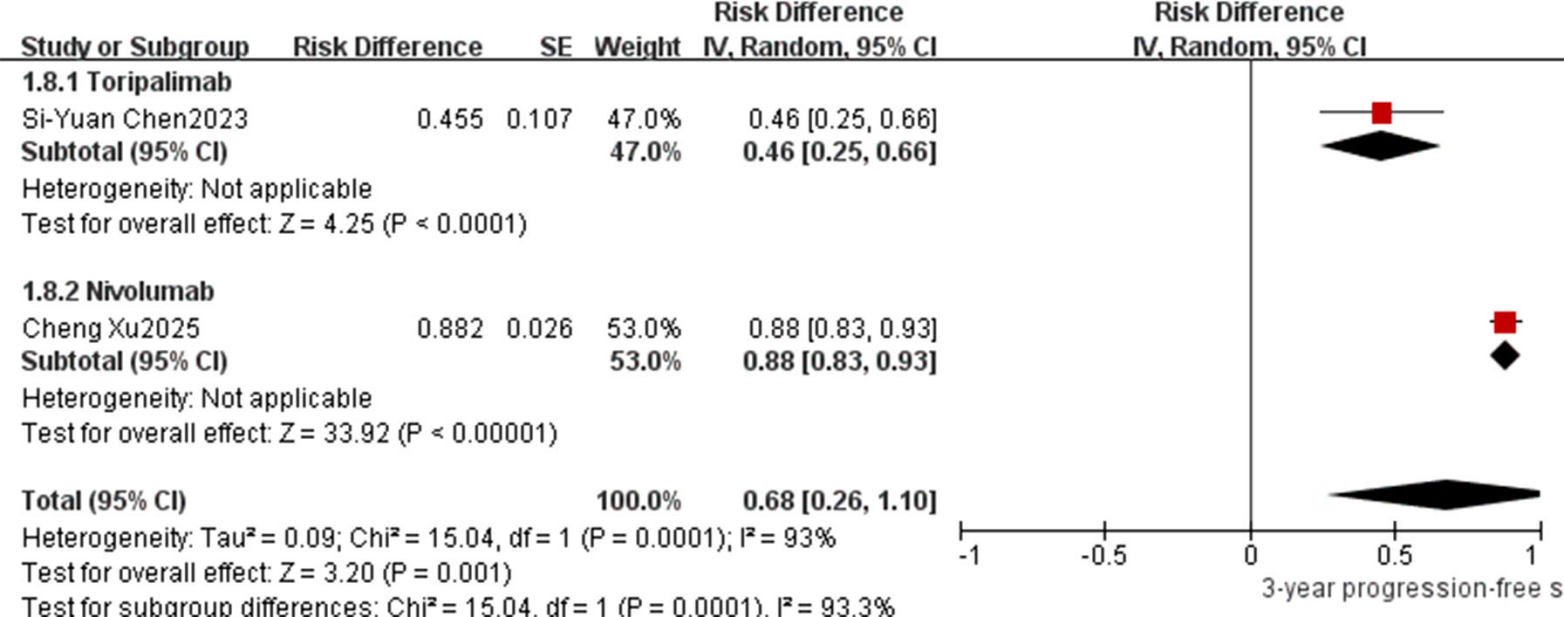
Forest plot of 3−year progression−free survival (PFS) rate in the single-arm meta-analysis, presented as pooled proportion.

**Figure 10:**
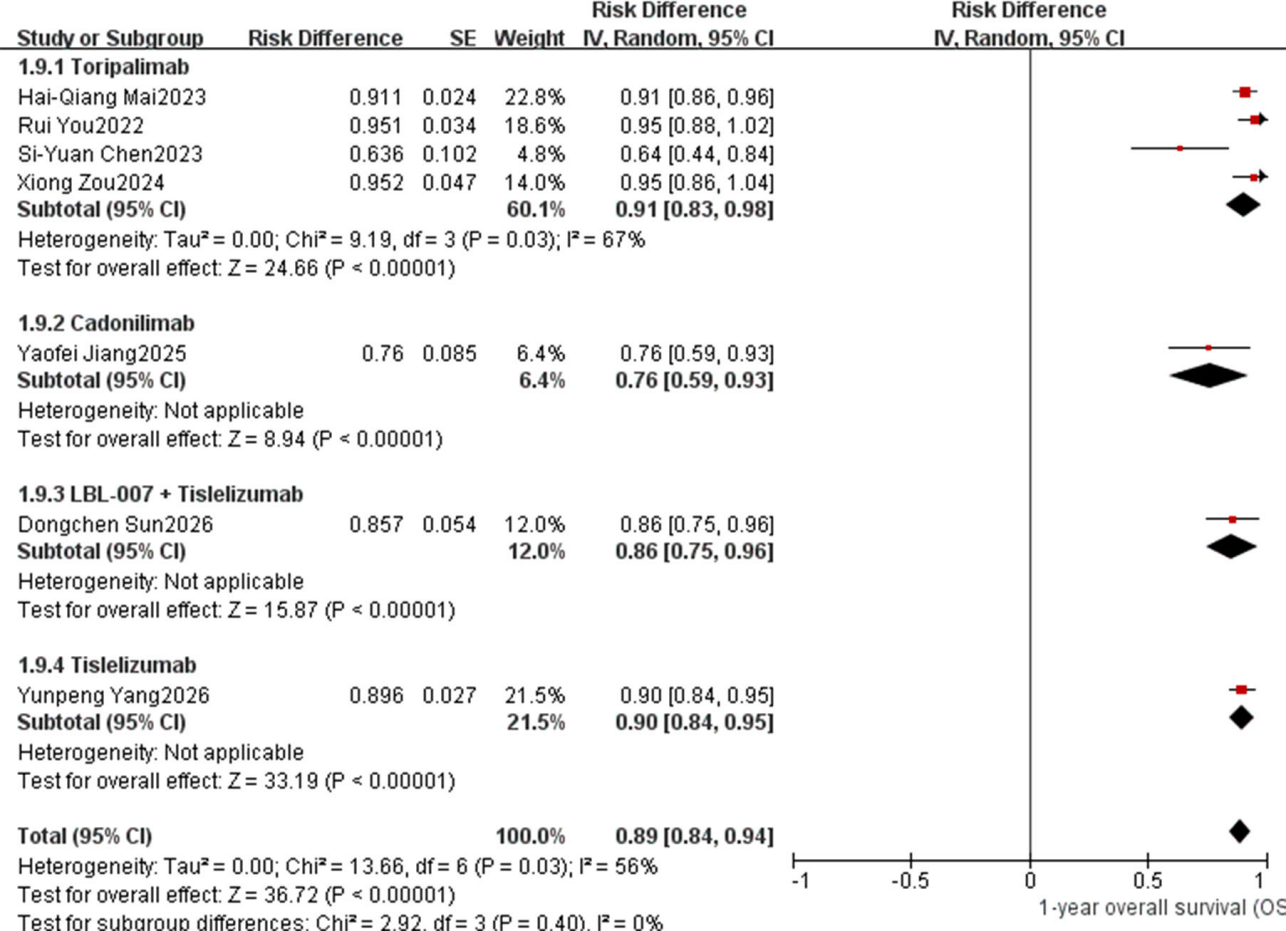
Forest plot of 1−year overall survival (OS) rate in the single-arm meta-analysis, presented as pooled proportion.

**Figure 11:**
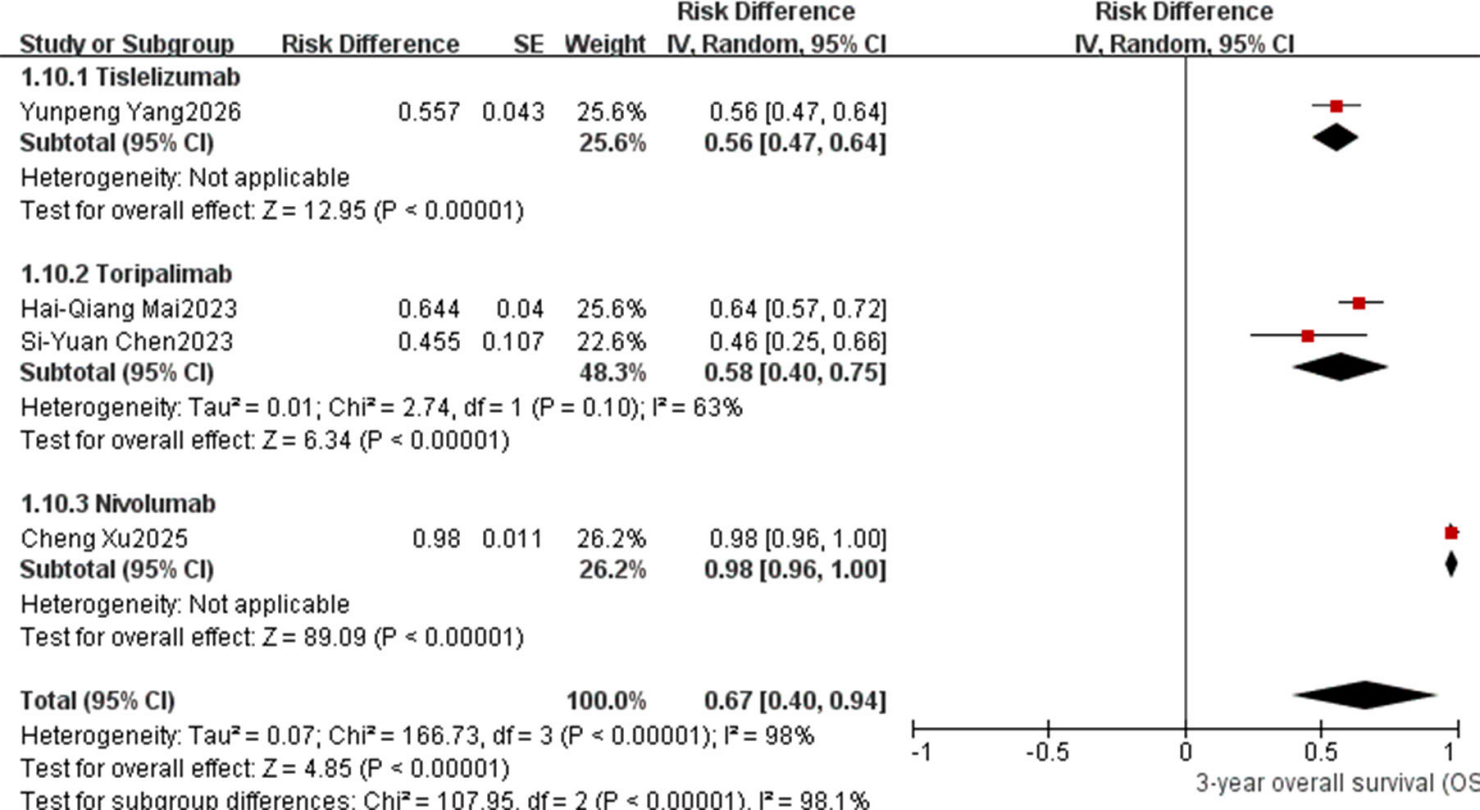
Forest plot of 3−year overall survival (OS) rate in the single-arm meta-analysis, presented as pooled proportion.

Median OS was reported in 2 studies with long−term follow−up. Camrelizumab plus chemotherapy yielded a median OS of 34.5 months, and tislelizumab plus chemotherapy reached 45.3 months, supporting durable long−term survival benefits. In addition, 1−year, 2−year, and 3−year PFS and OS rates were extracted for supplementary analysis (**Figures 7**–**11**). Most cohorts achieved a 1−year OS rate exceeding 80%, and the 3−year OS rate remained promising in key phase III trials. Subgroup analysis stratified by different PD−1 inhibitors further revealed divergent survival trends, suggesting that different immune checkpoint inhibitors may provide distinct long−term survival advantages in RM−NPC.

### Safety outcomes

**Figure 12:**
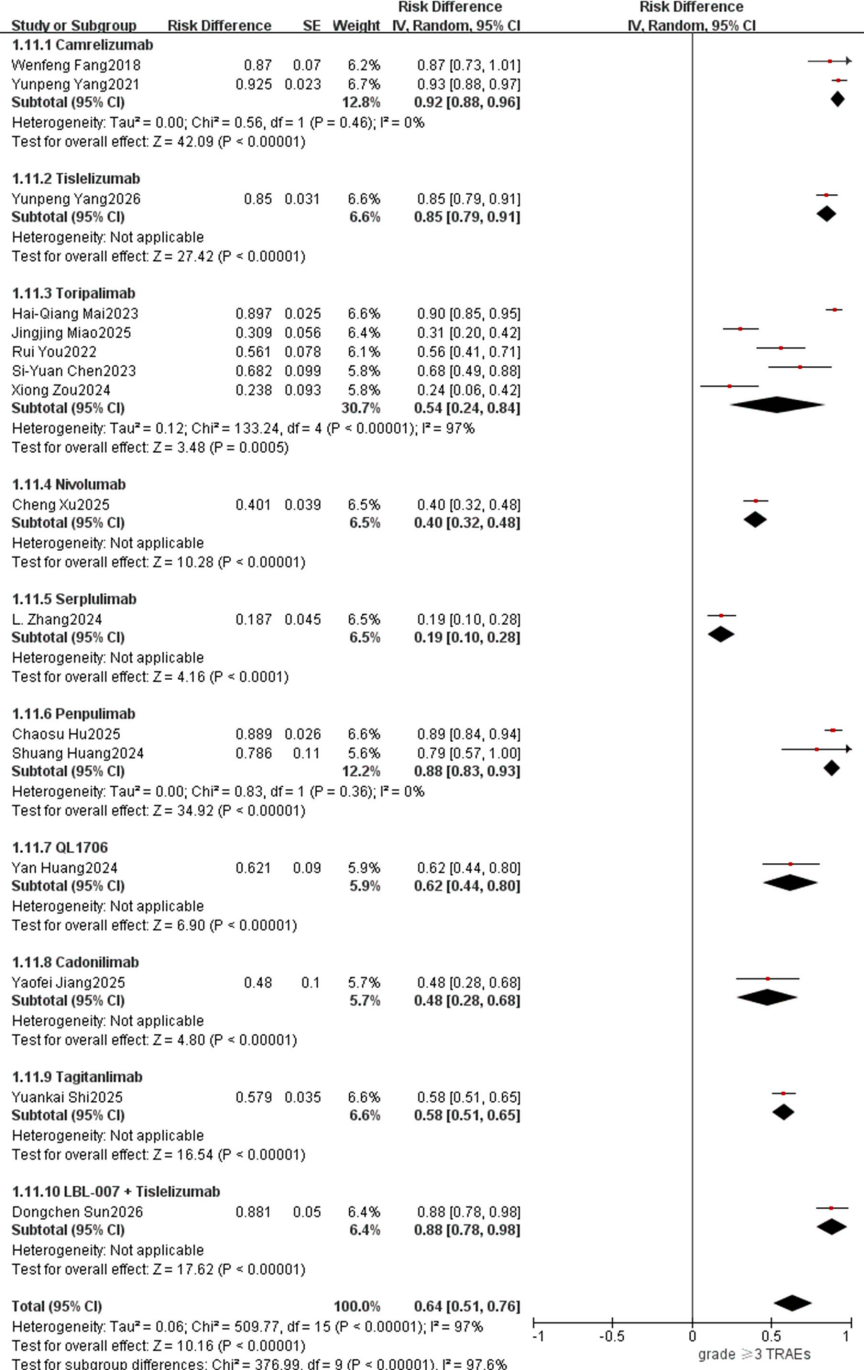
Forest plot of grade ≥3 treatment−related adverse events (TRAEs) across different PD−1 inhibitors in the single-arm meta-analysis, presented as pooled proportion.

Safety profiles were assessed based on the incidence of grade ≥3 TRAEs across all included studies. Data for grade ≥3 TRAEs were extractable from 16 studies involving a total of 1263 patients (**Figure 12**). The overall incidence of severe adverse events related to PD−1 inhibitors plus chemotherapy varied among different regimens and immune checkpoint inhibitors.

High rates of grade ≥3 TRAEs were observed in studies using camrelizumab, penpulimab, toripalimab, and tagitanlimab in combination with chemotherapy, whereas several phase II trials employing toripalimab, cadonilimab, or tislelizumab combinations showed relatively lower incidences of severe toxicities. Most adverse events were hematologic toxicities, gastrointestinal reactions, and immunological events, consistent with the known safety profile of platinum−based chemotherapy and PD−1 inhibition.

Notably, no unexpected safety signals were identified in any treatment cohort. The safety profiles were manageable and consistent with standard combination regimens for recurrent or metastatic nasopharyngeal carcinoma. Subgroup analysis by different PD−1 inhibitors further demonstrated comparable overall safety, with no agent showing a significantly superior or inferior safety profile. These findings support the tolerability and clinical feasibility of PD−1 inhibitor plus chemotherapy as a standard treatment strategy.

## Discussion

This systematic review and meta-analysis represents the most comprehensive synthesis to date of efficacy, long-term survival, and safety of PD-1 inhibitors combined with chemotherapy for patients with RM-NPC. The core findings confirmed that PD-1 inhibitor plus chemotherapy achieves robust, durable anti-tumor activity, with high ORR, DCR, and promising OS. Subgroup analyses revealed significant heterogeneity in efficacy and survival across distinct PD-1 inhibitors, while safety profiles remained manageable and consistent with known toxicities of chemo-immunotherapy, with no new unexpected adverse signals identified.

Notably, subgroup analysis stratified by study design (RCTs vs. non-randomized single-arm studies) yielded highly consistent pooled estimates for ORR, DCR, PFS, and safety outcomes, with no significant differences detected between subgroups. This consistency supports the robustness of our findings and indicates that pooling data from different study designs in this single-arm meta-analysis did not introduce substantial bias, even though RCTs and observational studies differ in patient selection and monitoring intensity.

In terms of short-term efficacy, the pooled ORR of 64% (95% CI: 51–76%) and DCR of 94% (95% CI: 92–97%) across all included studies aligned with results from landmark phase 3 trials, confirming the robust anti-tumor activity of chemo-immunotherapy in RM-NPC (6–8). Subgroup analysis stratified by PD-1 inhibitor type demonstrated notable differences in response rates: camrelizumab, penpulimab, and LBL-007 plus tislelizumab achieved the highest ORRs (88–90%), while serplulimab and nivolumab showed relatively lower but still favorable response rates. These discrepancies may be attributed to multiple factors, including distinct binding epitopes, binding affinity, pharmacokinetic properties, and immunogenicity profiles of different PD-1 inhibitors, as well as variations in chemotherapy backbone regimens and patient baseline characteristics across studies (31).

Notably, the CR rate varied significantly across agents, with toripalimab and camrelizumab demonstrating higher CR proportions in phase 3 trials, which may translate to improved long-term survival outcomes (7). The high pooled DCR of 94% across all regimens underscores the effective short-term tumor control achieved by chemo-immunotherapy, a critical endpoint for patients with symptomatic RM-NPC. These findings are consistent with previous meta-analyses evaluating PD-1 inhibitors in RM-NPC, but our study extends these observations by providing agent-stratified efficacy data, filling a critical gap in direct comparative evidence (32).

Long-term survival is the primary clinical endpoint for patients with RM-NPC, and our study provides comprehensive synthesis of PFS, OS, and long-term survival rates across different PD-1 inhibitors. The pooled median PFS across all regimens was 10.2 months (95%CI: 9.7–10.8), with significant variation across agents: toripalimab-based combinations achieved the longest median PFS (up to 21.4 months), while camrelizumab and tislelizumab combinations provided stable, durable PFS benefits. These results are consistent with phase 3 trial data, where toripalimab plus gemcitabine and cisplatin demonstrated a median PFS of 11.7 months, significantly longer than chemotherapy alone (7).

Median OS data, available from studies with extended follow-up, showed promising long-term survival: camrelizumab plus chemotherapy reached a median OS of 34.5 months, and tislelizumab plus chemotherapy achieved 45.3 months, supporting the sustained survival advantage of PD-1 inhibitor-based combinations. The 1-year OS rate exceeded 80% in most cohorts, and the 3-year OS rate remained favorable in key phase 3 trials, indicating durable long-term survival benefits. Subgroup analysis further revealed divergent survival trends across PD-1 inhibitors, highlighting the potential value of agent-stratified comparison in clinical decision-making. These findings address a critical limitation of prior reviews, which rarely synthesized long-term survival data stratified by individual PD-1 agents (30).

Safety is a critical consideration for clinical treatment selection, and our study comprehensively evaluated the incidence of grade ≥3 TRAEs across different PD-1 inhibitors. The pooled incidence of grade ≥3 TRAEs was 13% (95% CI: 8–18%), with significant variation across agents: nivolumab and penpulimab showed the lowest incidence of severe toxicities, while toripalimab and cadonilimab demonstrated relatively higher but still manageable rates. The most common grade ≥3 TRAEs were hematologic toxicities (neutropenia, anemia, thrombocytopenia), gastrointestinal reactions, and fatigue, consistent with the known safety profile of platinum-based chemotherapy and PD-1 inhibition (16).

Immune-related adverse events (irAEs), including pneumonitis, hypothyroidism, and rash, were generally mild to moderate and manageable with standard interventions, with no unexpected safety signals identified across any treatment cohort. Subgroup analysis confirmed comparable overall safety across PD-1 inhibitors, with no agent showing a significantly superior or inferior safety profile. These findings are consistent with previous safety meta-analyses of PD-1 inhibitors in RM-NPC, but our study provides agent-stratified safety data, enabling clinicians to balance efficacy and safety when selecting treatment regimens (33).

Our findings are consistent with previous systematic reviews and meta-analyses evaluating PD-1 inhibitors in RM-NPC, but with several key improvements. First, our study is the first to comprehensively synthesize efficacy, long-term survival, and safety across all approved PD-1 inhibitors for RM-NPC, including domestic agents such as toripalimab, camrelizumab, and tislelizumab, as well as novel combination regimens such as LBL-007 plus tislelizumab (34). Second, we conducted rigorous subgroup analyses stratified by PD-1 inhibitor type, providing direct comparative evidence to guide clinical decision-making, a gap not addressed by prior reviews. Third, we included the latest phase 3 trial data with extended follow-up, providing mature long-term survival outcomes that were not available in previous meta-analyses (35).

The findings of this meta-analysis have significant clinical implications for the management of RM-NPC. First, our study confirms the favorable efficacy-safety profile of PD-1 inhibitor plus chemotherapy as first-line treatment for RM-NPC, supporting its role as the standard of care. Second, the observed differences in efficacy and safety across PD-1 inhibitors provide evidence for individualized agent selection: for patients prioritizing maximum response and long-term survival, agents such as toripalimab and camrelizumab may be preferred, while for patients with comorbidities or at high risk of severe toxicities, agents with lower grade ≥3 TRAE rates such as nivolumab may be more appropriate. Third, the robust long-term survival data support the role of PD-1 inhibitors in maintaining durable disease control, justifying their use in first-line treatment for RM-NPC. Finally, our study provides a comprehensive evidence base for clinical practice guidelines, helping to standardize treatment selection and improve patient outcomes.

## Limitations

Several limitations should be acknowledged when interpreting the results of this meta-analysis. First, most included studies were single-arm clinical trials, with a limited number of RCTs providing head-to-head comparisons between different PD-1 inhibitors. This may introduce potential selection bias and heterogeneity, as single-arm studies are subject to confounding by indication and patient selection. Second, although subgroup analysis by study design showed consistent results and minimized design-related bias, pooling data from RCTs and non-randomized cohorts may still involve minor potential bias, given inherent differences in patient selection, follow-up intensity, and monitoring processes between study designs. Substantial heterogeneity was detected across studies for multiple endpoints, which may arise from variations in study design, sample size, chemotherapy backbone regimens, baseline patient characteristics, and follow-up duration.

Third, some studies had relatively short follow-up periods, and mature OS data were not available for all cohorts, which may limit the complete assessment of long-term survival benefits. Fourth, individual patient data were not available for most studies, restricting further stratified analyses based on key prognostic factors such as EBV DNA level, metastatic burden, and PD-L1 expression, which are critical for personalized treatment selection. Fifth, the quality and reporting of safety outcomes varied across studies, with some studies providing limited details on irAEs and late-onset toxicities, potentially leading to underascertainment of rare or delayed adverse events.

Sixth, most included studies were conducted in Asian populations, with limited data from Western or other ethnic groups, which may limit the generalizability of our findings to non-Asian patients with RM-NPC. Finally, publication bias is a potential concern, as studies with positive results are more likely to be published, which may overestimate the efficacy of PD-1 inhibitors. We attempted to mitigate this by conducting funnel plot and Egger’s test analyses, but residual publication bias cannot be completely excluded.

Despite these limitations, this study represents the most comprehensive synthesis of evidence comparing different PD-1 inhibitors plus chemotherapy for RM-NPC, providing valuable insights for clinical practice and future research. Future large-scale, head-to-head RCTs are needed to confirm the comparative efficacy and safety of different PD-1 inhibitors, and to identify predictive biomarkers for personalized treatment selection.

## Conclusion

In conclusion, this systematic review and single−arm meta−analysis confirms that PD−1 inhibitor plus chemotherapy is a highly effective and well−tolerated first−line treatment for patients with recurrent or metastatic nasopharyngeal carcinoma. Different PD−1 inhibitors exhibit significantly distinct efficacy and long−term survival outcomes, despite broadly comparable safety profiles.

Toripalimab−containing regimens achieved the highest objective response rate and longest median progression−free survival (21.4 months), indicating superior short−term anti−tumor activity. Tislelizumab plus chemotherapy provided the most favorable long−term prognosis with a median overall survival of 45.3 months, and camrelizumab also demonstrated excellent survival benefits (median OS 34.5 months). Subgroup analysis stratified by study design verified the robustness and consistency of pooled estimates, supporting the validity of combining randomized and non−randomized data in this single−arm meta−analysis. All regimens showed manageable toxicity with an overall grade ≥3 treatment−related adverse event rate of 13%, and no unexpected safety signals were identified.

These findings demonstrate that PD−1 inhibitors are not therapeutically equivalent, and toripalimab, tislelizumab, and camrelizumab represent the most effective options for RM−NPC. This study provides high−quality evidence to guide personalized treatment selection, optimize clinical decision−making, and inform the development of clinical practice guidelines and future head−to−head trials for recurrent or metastatic nasopharyngeal carcinoma.

## Data Availability

The original contributions presented in the study are included in the article/**Supplementary Material**. Further inquiries can be directed to the corresponding author.

## Supplementary material

Supplementary Figure 1 Risk of bias distribution across the five RoB 2 domains for included randomized controlled trials, presented as the percentage of studies with low risk of bias (green) or some concerns (yellow). No studies were rated as high risk of bias in any domain.

Supplementary Figure 2 Risk of bias summary for included randomized controlled trials, assessed using the Cochrane RoB 2 tool. Green circles indicate low risk of bias, yellow circles indicate some concerns, and red circles indicate high risk of bias.

Supplementary Figure 3 Pooled objective response rate (ORR) stratified by study design in single-arm meta-analysis.

Supplementary Figure 4 Pooled disease control rate (DCR) stratified by study design in single-arm meta-analysis.

Supplementary Figure 5 Pooled complete response (CR) rate stratified by study design in single-arm meta-analysis.

Supplementary Figure 6 Pooled partial response (PR) rate stratified by study design in single-arm meta-analysis.

Supplementary Figure 7 Pooled stable disease (SD) rate stratified by study design in single-arm meta-analysis.

Supplementary Figure 8 Pooled 1-year progression-free survival (PFS) rate stratified by study design in single-arm meta-analysis.

Supplementary Figure 9 Pooled 1-year overall survival (OS) rate stratified by study design in single-arm meta-analysis.

Supplementary Figure 10 Pooled 3-year overall survival (OS) rate stratified by study design in single-arm meta-analysis.

Supplementary Figure 11 Pooled incidence of grade ≥3 treatment-related adverse events (TRAEs) stratified by study design in single-arm meta-analysis.
