# Supplementary figures and images for: Efficacy, long-term survival and safety of different PD-1 inhibitors plus chemotherapy in recurrent or metastatic nasopharyngeal carcinoma: a systematic review and meta-analysis
Source: Front Oncol. 2026 Jun 15;16:1849030. doi: 10.3389/fonc.2026.1849030 (PMC13310671; doi:10.3389/fonc.2026.1849030)

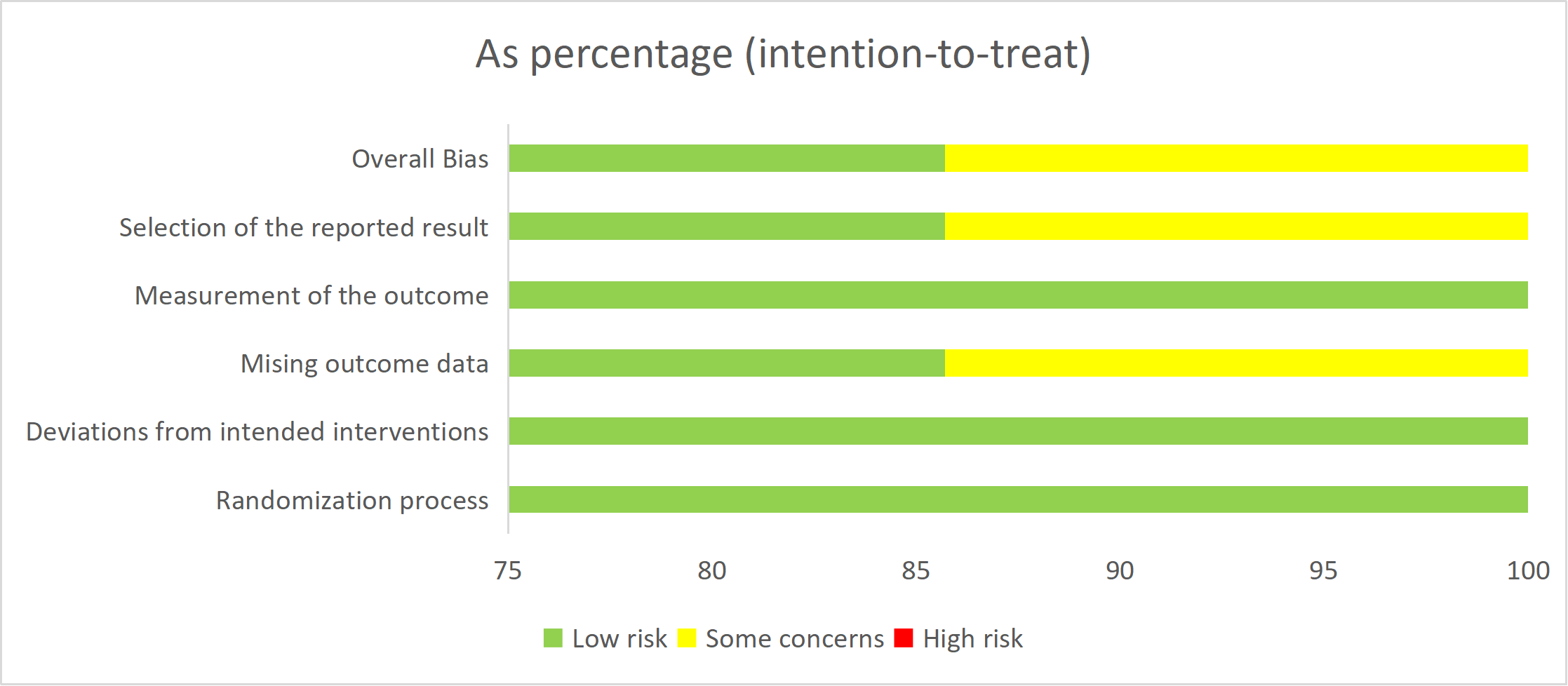

Supplement: Supplementary Figure 1 — Risk of bias distribution across the five RoB 2 domains for included randomized controlled trials, presented as the percentage of studies with low risk of bias (green) or some concerns (yellow). No studies were rated as high risk of bias in any domain. [file Image1.tif]

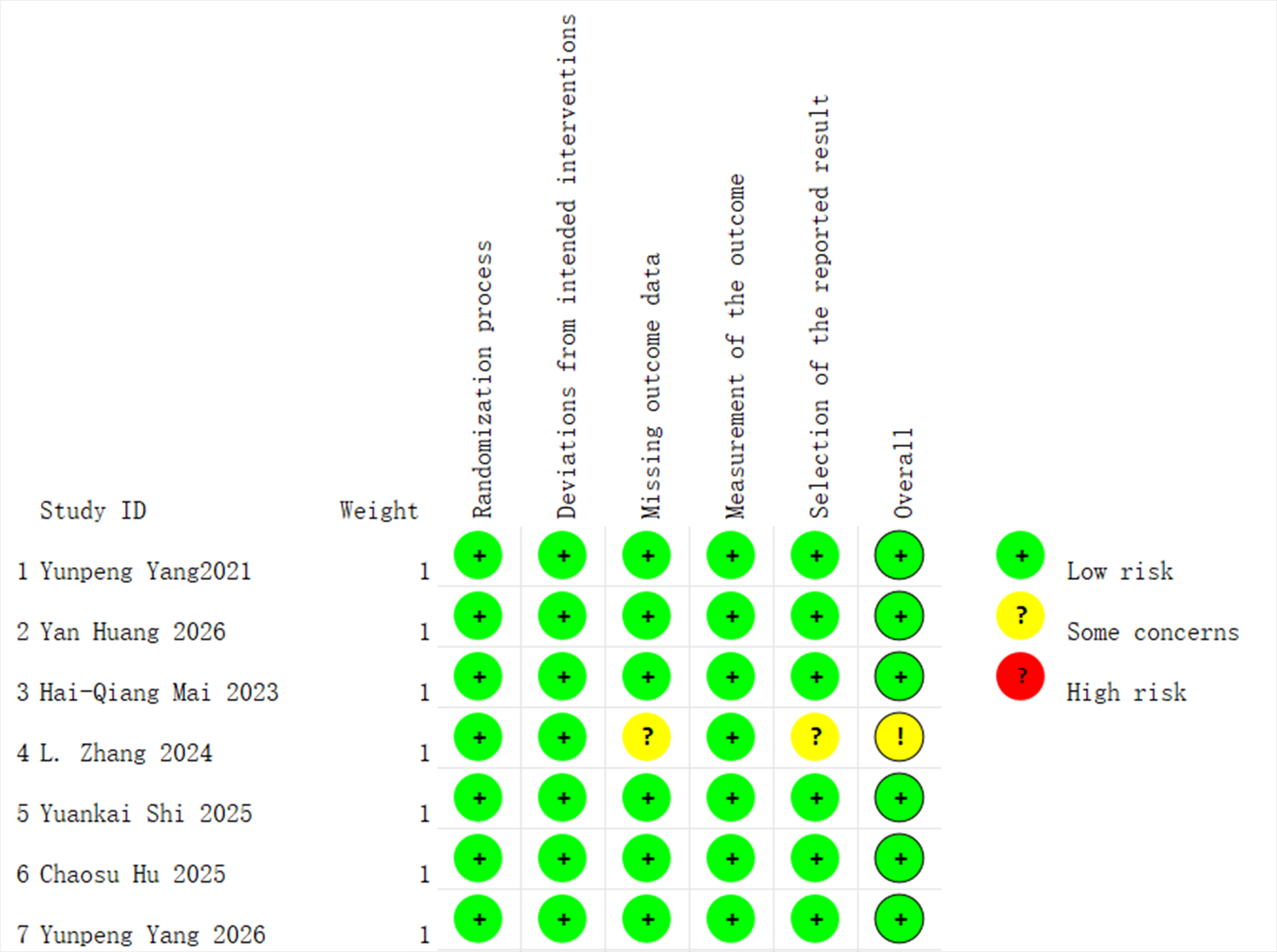

Supplement: Supplementary Figure 2 — Risk of bias summary for included randomized controlled trials, assessed using the Cochrane RoB 2 tool. Green circles indicate low risk of bias, yellow circles indicate some concerns, and red circles indicate high risk of bias. [file Image2.tif]

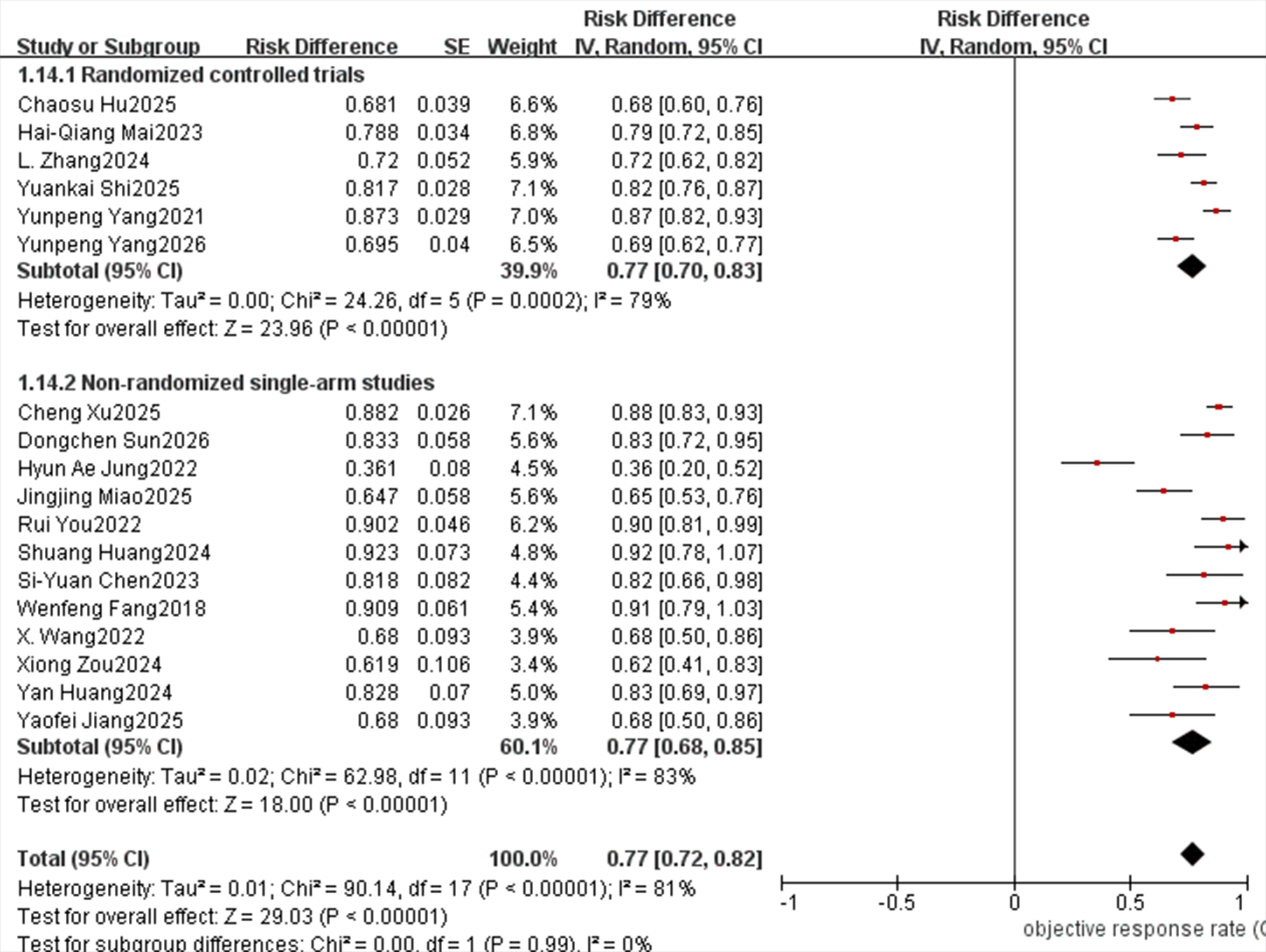

Supplement: Supplementary Figure 3 — Pooled objective response rate (ORR) stratified by study design in single-arm meta-analysis. [file Image3.tif]

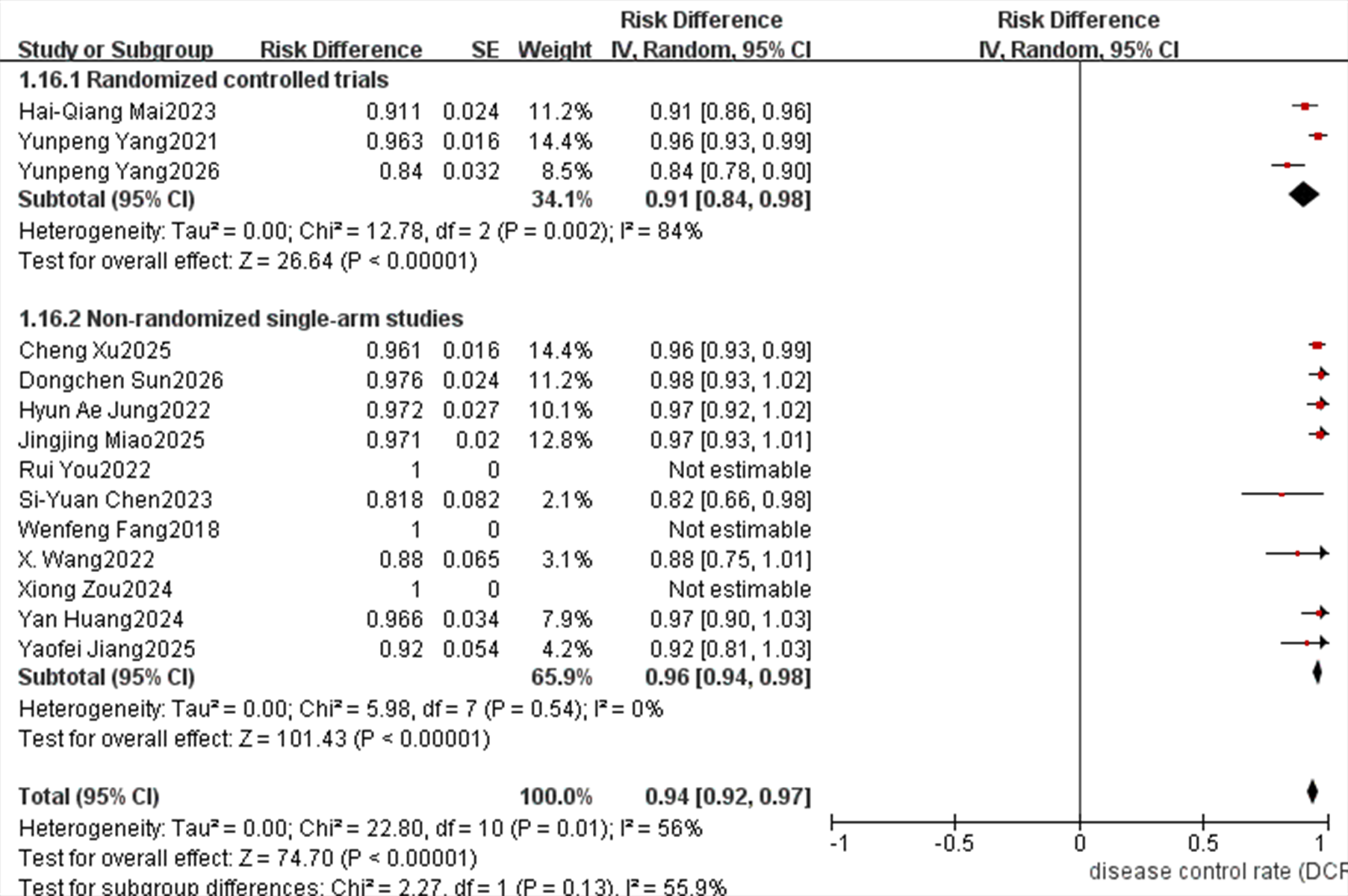

Supplement: Supplementary Figure 4 — Pooled disease control rate (DCR) stratified by study design in single-arm meta-analysis. [file Image4.tif]

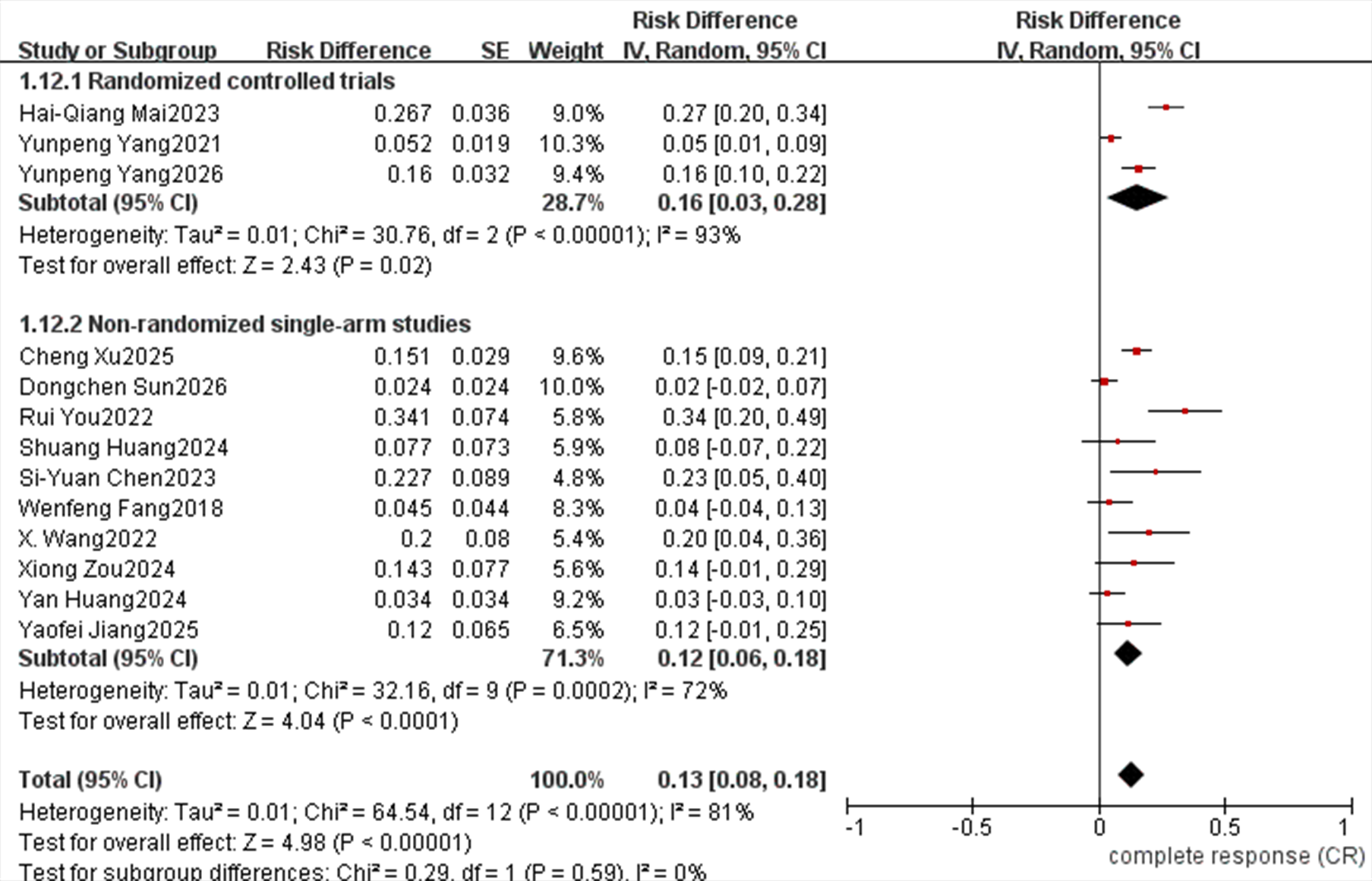

Supplement: Supplementary Figure 5 — Pooled complete response (CR) rate stratified by study design in single-arm meta-analysis. [file Image5.tif]

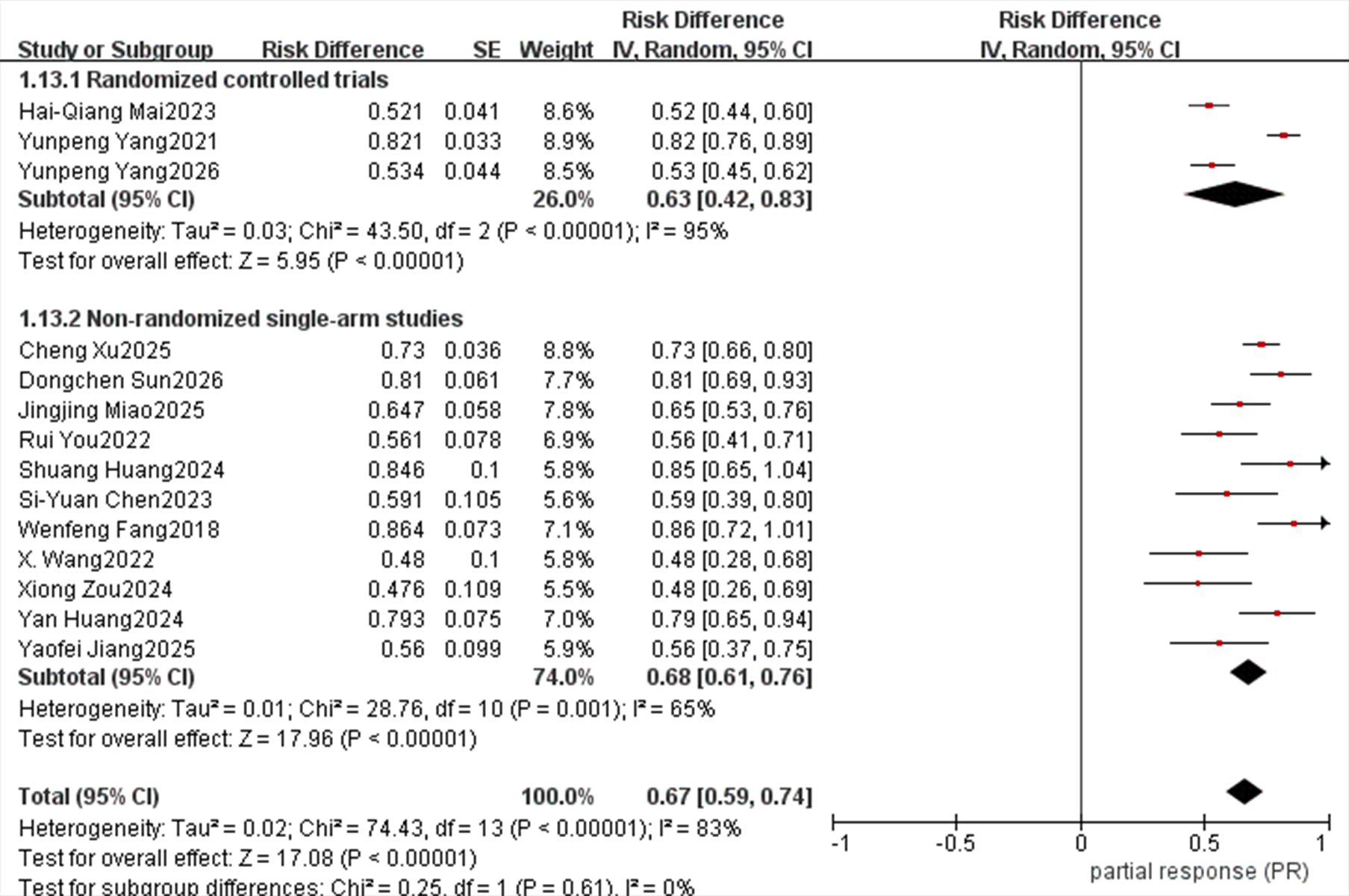

Supplement: Supplementary Figure 6 — Pooled partial response (PR) rate stratified by study design in single-arm meta-analysis. [file Image6.tif]

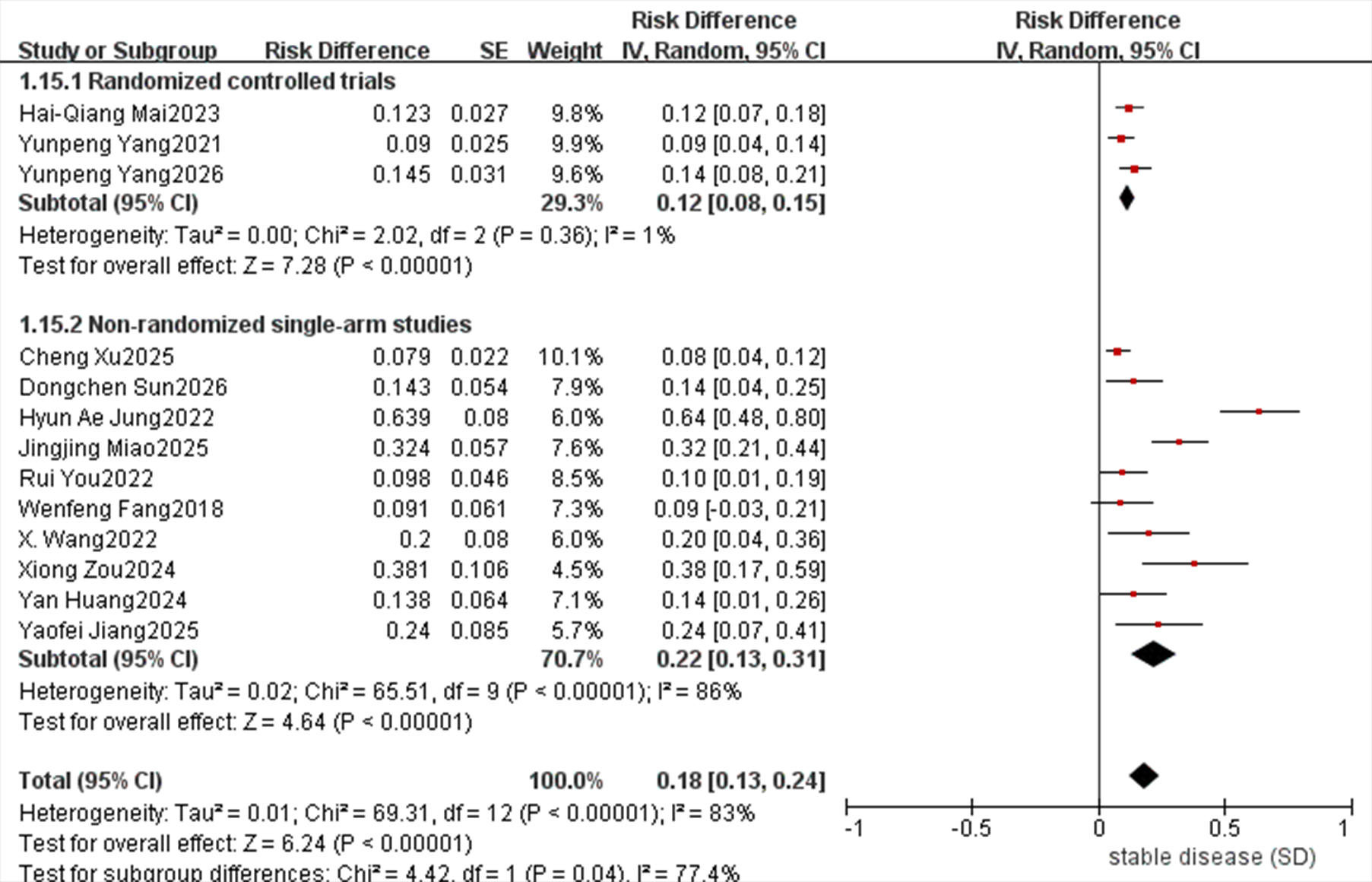

Supplement: Supplementary Figure 7 — Pooled stable disease (SD) rate stratified by study design in single-arm meta-analysis. [file Image7.tif]

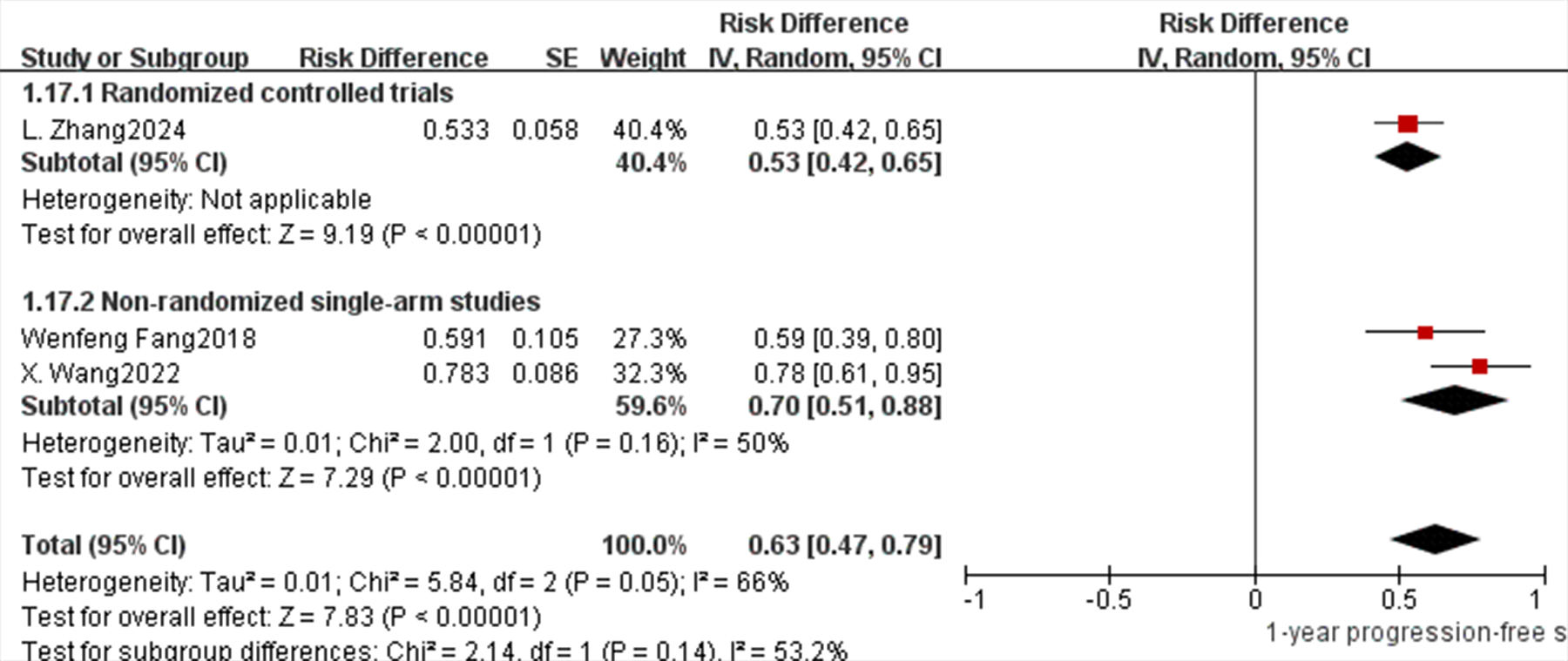

Supplement: Supplementary Figure 8 — Pooled 1-year progression-free survival (PFS) rate stratified by study design in single-arm meta-analysis. [file Image8.tif]

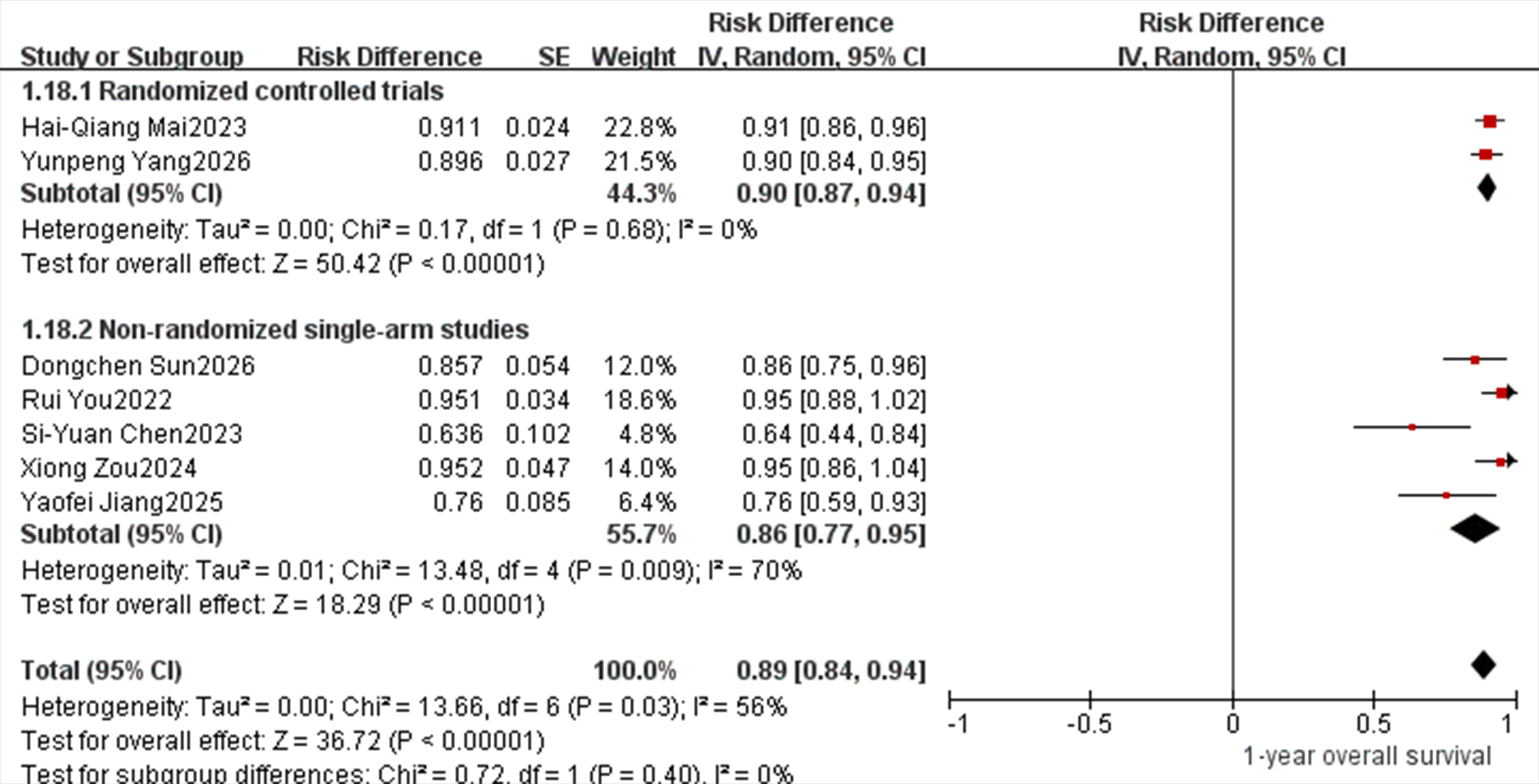

Supplement: Supplementary Figure 9 — Pooled 1-year overall survival (OS) rate stratified by study design in single-arm meta-analysis. [file Image9.tif]

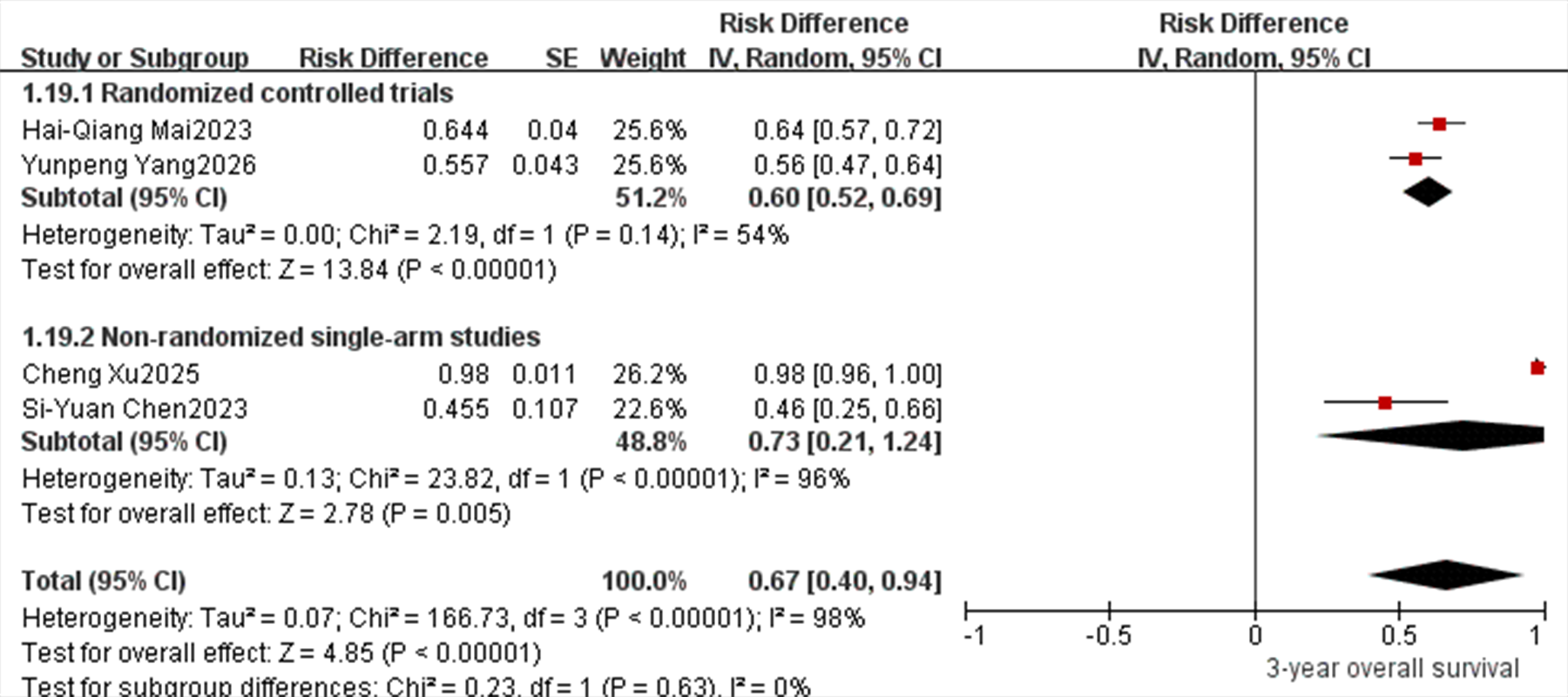

Supplement: Supplementary Figure 10 — Pooled 3-year overall survival (OS) rate stratified by study design in single-arm meta-analysis. [file Image10.tif]

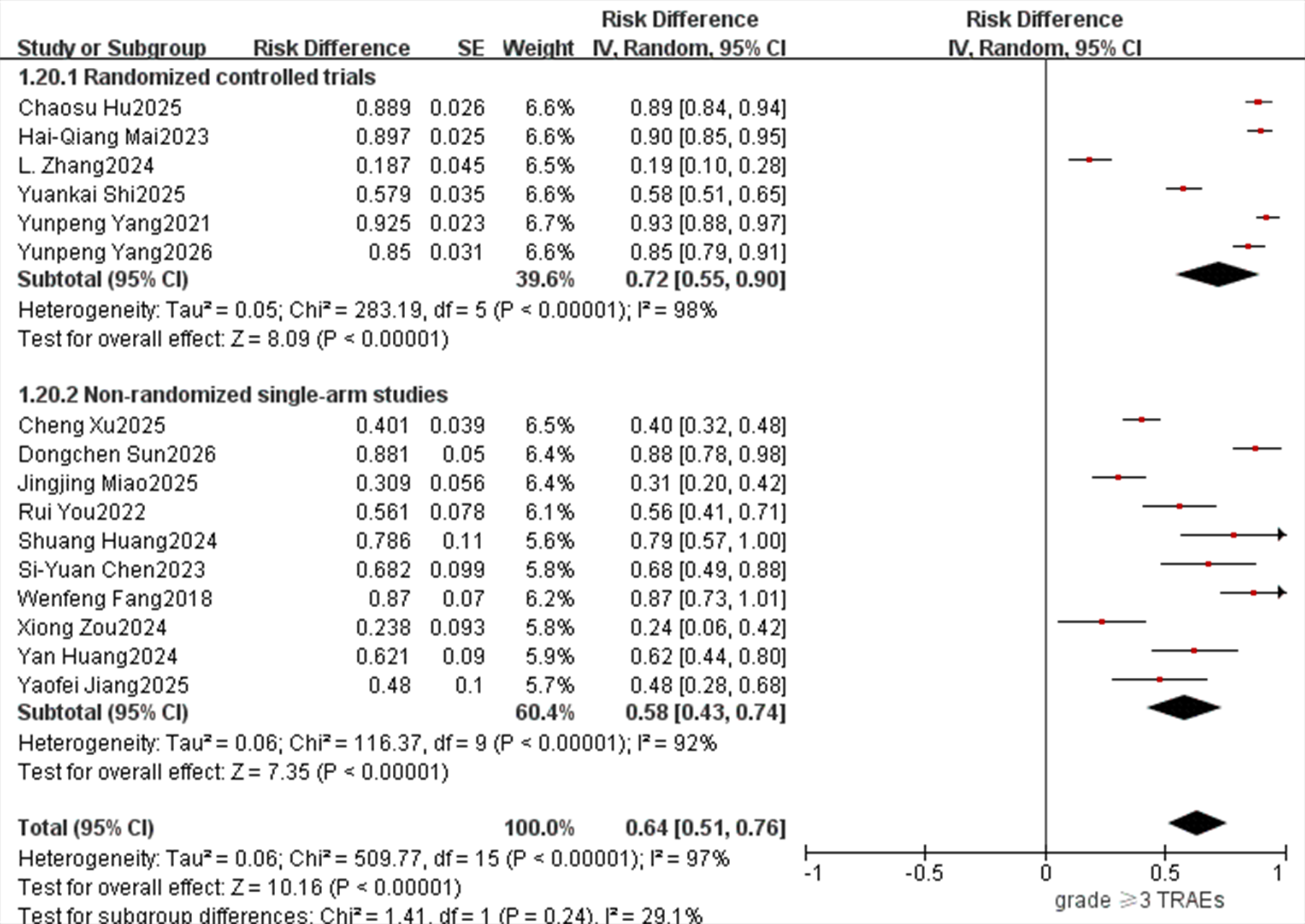

Supplement: Supplementary Figure 11 — Pooled incidence of grade ≥3 treatment-related adverse events (TRAEs) stratified by study design in single-arm meta-analysis. [file Image11.tif]
