# Supplementary material for: Efficacy, long-term survival and safety of different PD-1 inhibitors plus chemotherapy in recurrent or metastatic nasopharyngeal carcinoma: a systematic review and meta-analysis
Source: Front Oncol. 2026 Jun 15;16:1849030. doi: 10.3389/fonc.2026.1849030 (PMC13310671; doi:10.3389/fonc.2026.1849030)
Supplement: Supplementary file 12 [file DataSheet1.docx]

**Pubmed**

((nasopharyngeal carcinoma) OR (NPC)) AND ((recurrent) OR (metastatic)) AND ((PD-1 inhibitor) OR (anti-PD-1) OR (toripalimab) OR (tislelizumab) OR (camrelizumab) OR (nivolumab) OR (serplulimab) OR (penpulimab) OR (QL1706) OR (cadonilimab) OR (tagitanlimab)) AND (chemotherapy) AND (efficacy OR survival OR safety OR meta-analysis OR systematic review OR clinical trial)

**Embase**

('nasopharyngeal carcinoma'/exp OR 'nasopharyngeal carcinoma') AND ('recurrent disease'/exp OR 'metastasis'/exp) AND ('PD-1 inhibitor'/exp OR 'anti-PD-1 antibody'/exp OR toripalimab OR tislelizumab OR camrelizumab OR nivolumab OR serplulimab OR penpulimab OR QL1706 OR cadonilimab OR tagitanlimab) AND ('chemotherapy'/exp) AND ('efficacy'/exp OR 'survival'/exp OR 'safety'/exp OR 'meta-analysis'/exp OR 'systematic review'/exp OR 'clinical trial'/exp)

**Cochrane Library**

(nasopharyngeal carcinoma OR NPC) AND (recurrent OR metastatic) AND (PD-1 inhibitor OR anti-PD-1 OR toripalimab OR tislelizumab OR camrelizumab OR nivolumab OR serplulimab OR penpulimab OR QL1706 OR cadonilimab OR tagitanlimab) AND chemotherapy AND (efficacy OR survival OR safety OR meta-analysis OR systematic review OR clinical trial)

**Web of Science**

TS=((nasopharyngeal carcinoma) OR (NPC)) AND TS=((recurrent) OR (metastatic)) AND TS=((PD-1 inhibitor) OR (anti-PD-1) OR (toripalimab) OR (tislelizumab) OR (camrelizumab) OR (nivolumab) OR (serplulimab) OR (penpulimab) OR (QL1706) OR (cadonilimab) OR (tagitanlimab)) AND TS=(chemotherapy) AND TS=(efficacy OR survival OR safety OR meta-analysis OR systematic review OR clinical trial)
