# Supplementary material for: Efficacy, long-term survival and safety of different PD-1 inhibitors plus chemotherapy in recurrent or metastatic nasopharyngeal carcinoma: a systematic review and meta-analysis
Source: Front Oncol. 2026 Jun 15;16:1849030. doi: 10.3389/fonc.2026.1849030 (PMC13310671; doi:10.3389/fonc.2026.1849030)
Supplement: Supplementary file 13 [file DataSheet2.docx]

**Supplementary Table 1** Assessment of non-randomized controlled trials in the version of MINORS.

| Study | A clearly  stated  aim | Inclusion of  consecutive  patients | Prospective  collection of  data | Endpoint  appropriate  to the study  aim | Unbiased  assessment  of endpoints | Follow-up  period  appropriate  to the major  endpoint | Loss to  follow  up not  exceeding  5% | Prospective  calculation  of the study  size | Total score |
| --- | --- | --- | --- | --- | --- | --- | --- | --- | --- |
| Wenfeng Fang2018 | 2 | 2 | 2 | 2 | 2 | 1 | 2 | 2 | 15 |
| X. Wang2022 | 2 | 2 | 2 | 2 | 2 | 2 | 1 | 2 | 15 |
| Rui You2022 | 2 | 2 | 2 | 2 | 2 | 1 | 1 | 1 | 13 |
| Hyun Ae Jung2022 | 2 | 2 | 2 | 2 | 2 | 1 | 2 | 2 | 15 |
| Si-Yuan Chen2023 | 2 | 2 | 2 | 2 | 2 | 2 | 1 | 2 | 15 |
| Shuang Huang2024 | 2 | 2 | 2 | 2 | 2 | 2 | 2 | 2 | 16 |
| Yan Huang2024 | 2 | 2 | 2 | 1 | 2 | 1 | 2 | 2 | 14 |
| Xiong Zou2024 | 2 | 2 | 2 | 2 | 2 | 1 | 2 | 2 | 15 |
| Yaofei Jiang2025 | 2 | 2 | 2 | 2 | 2 | 2 | 1 | 2 | 15 |
| Jingjing Miao2025 | 2 | 2 | 2 | 1 | 1 | 1 | 1 | 1 | 11 |
| Cheng Xu2025 | 2 | 2 | 2 | 2 | 2 | 2 | 1 | 2 | 15 |
| Dongchen Sun2026 | 2 | 2 | 2 | 2 | 2 | 2 | 2 | 2 | 16 |
